# Early-life exposure to perfluoroalkyl substances in relation to serum adipokines in a longitudinal birth cohort

**DOI:** 10.1016/j.envres.2021.111905

**Published:** 2021-08-19

**Authors:** Yu-Hsuan Shih, Annelise J. Blomberg, Louise Helskov Jørgensen, Pál Weihe, Philippe Grandjean

**Affiliations:** a Department of Environmental Health, Harvard T.H. Chan School of Public Health, Boston, MA, 02115, USA; b Division of Occupational and Environmental Medicine, Lund University, Lund, Sweden; c Department of Clinical Biochemistry and Pharmacology, Odense University Hospital and Institute of Clinical Research, University of Southern Denmark, Odense, Denmark; d Department of Occupational Medicine and Public Health, Faroese Hospital System, Torshavn, Faroe Islands; e Center of Health Science, University of the Faroe Islands, Torshavn, Faroe Islands; f Department of Environmental Medicine, University of Southern Denmark, Odense, Denmark

**Keywords:** Adipokine, Childhood, Metabolic health, Perfluoroalkyl substances, Prospective study

## Abstract

**Background:**

Per- and polyfluoroalkyl substances (PFAS) exposure has been linked to metabolic health outcomes such as obesity, and changes in adipokine hormones may be one of the underlying biological mechanisms. We prospectively evaluated the associations between prenatal and early childhood exposures to PFASs and adipokines in children.

**Material and methods:**

PFAS concentrations were measured in serum samples collected at birth, 18 months, and 5 and 9 years, and adiponectin, leptin, leptin receptor, and resistin were measured in serum samples collected at birth and 9 years. We used multivariable linear regression models to estimate the percent change in serum-adipokine concentrations for a doubling in serum-PFAS concentrations. The potential sex-specific effect of PFAS was assessed by including an interaction term between PFAS and sex in each model. Bayesian kernel machine regression (BKMR) was implemented to evaluate the overall effect of PFAS mixtures.

**Results:**

Significant associations with leptin, leptin receptor, and resistin at age 9 years were observed for serum-PFAS concentrations at 18 months and 5 and 9 years, whereas associations for PFAS concentrations at birth were mostly null. However, we observed a positive association between serum-PFHxS at birth and leptin receptor at birth. We found limited evidence regarding modification effect of sex on serum-PFAS concentrations. BKMR findings were consistent and suggested some significant effects of the overall PFAS mixtures at 18 months and 5 and 9 years on adipokine concentrations at 9 years.

**Conclusions:**

Given the associations of PFAS exposure with both adipokine hormones and metabolic functions, future studies should include assessment of adipokine hormones when examining PFAS-associated metabolic alterations.

## 1. Introduction

Perfluorinated alkyl substances (PFASs) are persistent synthetic compounds that have been widely produced since the 1940s due to their unique surfactant characteristics (ATSDR and AuthorAnonymous, 2021; EFSA 2020). The global use of this class of chemicals has led to widespread human exposure, and PFASs are now detectable in blood among general populations worldwide (Sunderland et al., 2019).

Early-life exposure to PFASs has been associated with metabolic health outcomes including an increased risk of obesity and diabetes later in life (ATSDR and AuthorAnonymous, 2021; Blomberg et al., 2021; EFSA CONTAM Panel (EFSA Panel on Contaminants in the Food Chain) et al., 2020; Karlsen et al., 2017). However, the underlying biological mechanism is still unclear. One of the potential mechanisms by which exposure to PFASs may affect metabolic health is by altering adipokine hormones (Du et al., 2018; Jung and Choi, 2014). Adipokine hormones are secreted by adipose tissue and play a key role in systemic metabolism and inflammation. For example, leptin is a hormone that regulates energy balance by controlling appetite through the central nervous system, and it absence has been associated with morbid obesity (Yang and Barouch, 2007). Adiponectin, an insulin sensitizer and an anti-inflammatory, is an important regulator of glucose and lipid homeostasis that has also been linked to obesity risk (Gariballa et al., 2019). Further, there is increasing evidence connecting metabolism, inflammatory, and autoimmune diseases to resistin, an adipocyte-specific hormone (Jamaluddin et al., 2012; Tripathi et al., 2020). However, few previous studies have examined the association between PFAS exposure and serum concentrations of adipokine hormones in children, and the majority of these studies focused on leptin and adiponectin only and reported inconsistent results (Ashley-Martin et al., 2017; Buck et al., 2018; Domazet et al., 2020; Fleisch et al., 2017; Minatoya et al., 2017; Shelly et al., 2019). Only two previous studies considering longitudinal effects of PFAS exposure (Fleisch et al., 2017; Shelly et al., 2019), and none of them implemented mixture methods to estimate the overall effect of exposures to PFAS mixtures.

Thus, the present birth cohort study aimed to evaluate prospectively the associations of serum-PFAS concentrations measured at birth, 18 months, and 5 and 9 years with adipokine hormones both at birth and at age 9 years. The present report refers to the second study of PFAS exposure and adipokine hormones in Faroese children. Compared to the first study within an older cohort born in 1997–2000 (Cohort 3) (Shelly et al., 2019), this study has a larger sample size and lower PFAS exposure levels. Because the previous prospective study reported sex-specific associations between PFAS and adipokines, we hypothesized that the associations explored in the present study could be sex-specific. In addition to linear regression models, Bayesian kernel machine regression was also implemented (BKMR) to evaluate the potential mixture effects of PFASs on adipokine hormones.

## 2. Material and Methods

### 2.1. Study population

Between October 2007 and April 2009, a birth cohort of 490 mother-child pairs was recruited from the National Hospital in Tórshavn in the Faroe Islands. The cohort was limited to singleton children born at term. We collected umbilical cord blood samples at birth immediately after clamping. When children reached ages 18 months and 5 and 9 years, mother-child pairs were invited back for follow-up clinical examinations including maternal questionnaires, child physical examination, and blood sample collection. More detailed information on this cohort can be found elsewhere (Grandjean et al., 2017; Weihe et al., 2016). PFAS concentrations were analyzed in serum samples collected at birth, 18 months, and 5 and 9 years. Concentrations of adipokine hormones were measured in serum samples collected at birth and 9 years. After excluding subjects who missed an examination or did not contribute a sufficient blood volume, 463 mother-child pairs were included for the analyses on adipokine and PFAS concentrations at birth, and 358, 277, 294, and 370 cohort members were included for the analyses on the associations of PFAS at birth, 18 months, and 5 and 9 years with adipokine at 9 years, respectively. The study protocol was approved by the Faroese ethical review committee and the Harvard T.H. Chan School of Public Health institutional review board. Written informed consent was obtained from all participating mothers.

### 2.2. PFAS analysis

We analyzed serum samples collected at birth, 18 months, and 5 and 9 years for concentrations of five common PFASs: perfluorooctane sulfonic acid (PFOS), perfluorooctanoic acid (PFOA), perfluorohexane sulfonic acid (PFHxS), perfluorononanoic acid (PFNA) and perfluorodecanoic acid (PFDA). We used online solid-phase extraction followed by high-pressure liquid chromatography with tandem mass spectrometry (Haug et al., 2009), with extraction conducted using a Thermo Scientific EQuan MAX system (Thermo Scientific, San Jose, CA). High precision was suggested by the within-batch coefficient of <3% and between-batch coefficients of 5–6%. Measurement accuracy was controlled by including standard reference material (e.g., NIST, 1957) in each batch. The laboratory regularly participates in the German External Quality Assessment Scheme (G-EQUAS) for analyses in biological materials and is certified by the Human Biomonitoring Monitoring program for the European Union (HBM4EU) for analysis pf PFASs. The limit of detection (LOD) for all PFASs was 0.03 ng/ml. For participants with values below the LOD, a value of 0.015 ng/ml was assigned.

### 2.3. Adipokine analysis

Serum concentrations of adipokine hormones at birth and age 9 years were measured using commercial ELISA kits according to the instructions from the manufacturer’s manuals. The following kits from Biovendor (Brno, Czech Republic) were used: leptin (VEN/RD191001100), leptin receptor (VEN/RD194002100), adiponectin (VEN/RD191023100), and resistin (VEN/RD191016100). For Adiponectin, all samples were diluted 1:1200 to obtain measurable levels. Pooled donor serum obtained from the Department of Clinical Immunology at Odense University Hospital was used to evaluate the performance of the kits. High accuracy was suggested by low coefficients of variation of 2.9% for leptin, 2.1% for leptin receptor, 5.3% for adiponectin, and 7.4% for resistin. We also calculated the ratios of leptin by leptin receptor (Lep/LepR ratio) and adiponection by leptin (Adpn/Lep ratio) as indicators of leptin resistance and adipose tissue inflammation, respectively (Fruhbeck et al., 2018, 2019; Herrick et al., 2016).

### 2.4. Covariates

Standard questionnaires were used to collect maternal age (years), maternal smoking during pregnancy (none, 1–5 cigarettes per day, >5 cigarettes per day), maternal education (low, medium, high), and nutritional habits (e.g., whale consumption) during pregnancy. We abstracted additional obstetric information such as parity (primiparous, multiparous) and pre-pregnancy weight, height, and body mass index (BMI in kg/m^2^) from hospital charts. Maternal and child information such as exclusive and mixed breastfeeding duration and child nutritional habits were collected through 18-month and 9-year maternal standard questionnaires. Child’s weight, height, and BMI were obtained at the clinical examinations.

### 2.5. Statistical analysis

Spearman’s rank correlation coefficients assessed pairwise within-visit and cross-visit PFAS correlations. We assessed differences in adipokine and PFAS concentrations by sex using the Wilcoxon rank-sum test.

To assess the association between serum-PFAS concentrations and adipokine hormones, we first performed multivariable linear regression to estimate a beta coefficient and its 95% confidence interval (CI) for each PFAS. PFAS concentrations were log_2_-transformed and adipokine concentrations and the two ratio indicators were natural log-transformed due to skewed distributions. The beta coefficient obtained was interpreted as the change in adipokine concentration for a doubling in PFAS concentration. We assessed statistical interactions by child sex by including an interaction term between PFAS and sex in the linear regression model. The corresponding value of the observed Wald test statistic was used to obtain the p-value for interaction (P_interaction_). We considered P_interaction_ < 0.1 as significant for interaction effects.

Potential confounders were selected and included in the regression model if they had been associated with prenatal and childhood PFAS concentrations and metabolic functions in prior literature (directed acyclic graphs are included as Figure S1 and S2) (Braun et al., 2016; Buck et al., 2018; Domazet et al., 2020; Fleisch et al., 2017; Halldorsson et al., 2012; Hoyer et al., 2015; Karlsen et al., 2017; Mora et al., 2017; Shelly et al., 2019). We included maternal education and child sex in all models. For models evaluating the associations with PFAS concentrations at birth, 18 months, and 5 years, we additionally adjusted for variables associated with maternal PFAS exposure, including maternal age, maternal pre-pregnancy BMI, maternal smoking during pregnancy, and parity.

We next applied a BKMR model to estimate the overall effect of exposure to the five PFASs at each point in time on each of the adipokine hormones measured at birth and age 9 years, and to identify the most important PFAS associated with each adipokine hormone (Bobb et al., 2015, 2018). The BKMR allows for potential nonlinear associations by utilizing a kernel function to specify the unknown PFAS-adipokine relationship. This non-parametric approach also allows for potential interactions between the five PFASs while adjusting for additional covariates of interest. In the current analysis, all serum-PFAS concentrations were log_2_-transformed and standardized. We applied a component-wise variable selection approach with Gaussian kernel function, and then we fitted the model by running 200,000 iterations of a Markov Chain Monte Carlo (MCMC) sampler with the first 100,000 as burn-in. The posterior inclusion probabilities (PIPs) were generated to assess the relative importance of each PFAS. Estimates of the exposure-outcome function for each PFAS-adipokine relationship were produced while fixing all other PFASs at their median. We further explored pairwise interactions between each pair of PFASs by plotting the exposure-outcome function for the first PFAS while fixing the second PFAS at its 25th, 50th, and 75th percentile. Lastly, the overall effect of PFAS mixtures was evaluated by comparing the difference in each adipokine hormone when all five PFASs were set to their specific percentiles (i.e., 10th to 90th in increments of 5), as compared to when they were all fixed at their 50th percentile. Sex-stratified BKMR models were conducted to estimate the potential sex-specific associations between PFAS mixtures and adipokine hormones. The analyses were performed using the R package “bkmr” version 0.2.0 (Bobb, 2017).

Several sensitivity analyses were conducted to assess the robustness of our primary findings. First, given the repeated PFAS measures for the same child, generalized estimating equations (GEEs) with a working independence correlation matrix were used to evaluate the associations of PFAS concentrations measured at four exposure periods (i.e., birth, 18 months, and 5 and 9 years) with serum-adipokine concentrations at 9 years (Pepe et al., 1999; Sanchez et al., 2011). We included a three-way interaction term between serum-PFAS concentrations, exposure period, and sex to allow the effect of PFAS to vary by exposure period and sex. We also adjusted for maternal education, maternal age, maternal pre-pregnancy BMI, maternal smoking during pregnancy, and parity in the GEE models. These analyses were conducted using the R package “geepack” version 1.3–2 (Højsgaard et al., 2005).

Second, since studies have shown that breastfeeding is associated with both PFAS exposure and metabolic health outcomes (Martin et al., 2005; Mogensen et al., 2015; Owen et al., 2005), we conducted a sensitivity analysis where we additionally evaluated models with PFAS concentrations at 18 months and 5 years in relation to adipokine concentrations at 9 years after including duration of exclusive breastfeeding (months) as a covariate. Additionally, PFAS exposures in this population appear to be elevated due to consumption of marine food (Eriksson et al., 2013; Weihe et al., 2008) that may impact serum-adipokine concentrations (Moreno-Aliaga et al., 2010). We performed another sensitivity analysis where we additionally adjusted for maternal whale consumption during pregnancy (yes, no) or child whale consumption at 9 years (yes, no) in the models. The potential confounding effect of maternal whale consumption was evaluated in models with serum-PFAS concentrations at birth, 18 months, and 5 years, and child whale consumption at 9 years was evaluated in models with serum-PFAS concentrations at 9 years.

## 3. Results

Major characteristics of the 463 mother-child pairs for analysis of PFAS concentrations and adipokine hormones at birth are shown in Table 1. Approximately equal numbers of boys and girls were enrolled, most of them with older siblings. While the majority of the mothers were non-smokers, about one fifth of the mothers consumed pilot whale during pregnancy. The average length of exclusive breastfeeding was four and a half months.

The distributions of the serum-PFAS concentrations, overall and by child sex, are summarized in Figure S3. All five PFAS concentrations at birth were lower than PFAS concentrations in childhood. Distributions of PFASs were in general similar between males and females, although males had higher PFOS (P_Wilcoxon_ = 0.01) and PFHxS (P_Wilcoxon_ < 0.001) concentrations at 9 years than females. Figure S4 shows within-visit and between-visit correlations of five PFASs. In general, within-visit correlations were higher for PFAS concentrations at 18 months than at birth and later childhood, with Spearman correlation coefficients from 0.65 between PFHxS and PFNA to 0.89 between PFNA and PFDA. The latter two were strongly correlated across all time points (Spearman’s rho = 0.77–0.89), as was PFOS and PFHxS (Spearman’s rho = 0.58–0.85). Considering associations between visits, concentrations at ages 18 months and 5 and 9 years were relatively highly correlated. In contrast, PFAS concentrations at birth showed low correlations with subsequent PFAS concentrations.

The distributions of adipokine hormones at birth and age 9 years are shown in Table 2. Compared to at birth, serum concentrations of adiponectin, leptin, and resistin were much lower at age 9 years, whereas leptin receptor was much higher at age 9 years. At both time points, females generally had higher serum concentrations of adiponectin, leptin, and resistin, but lower concentrations of the leptin receptor.

### 3.1. Linear regressions

**Fig. 1.**
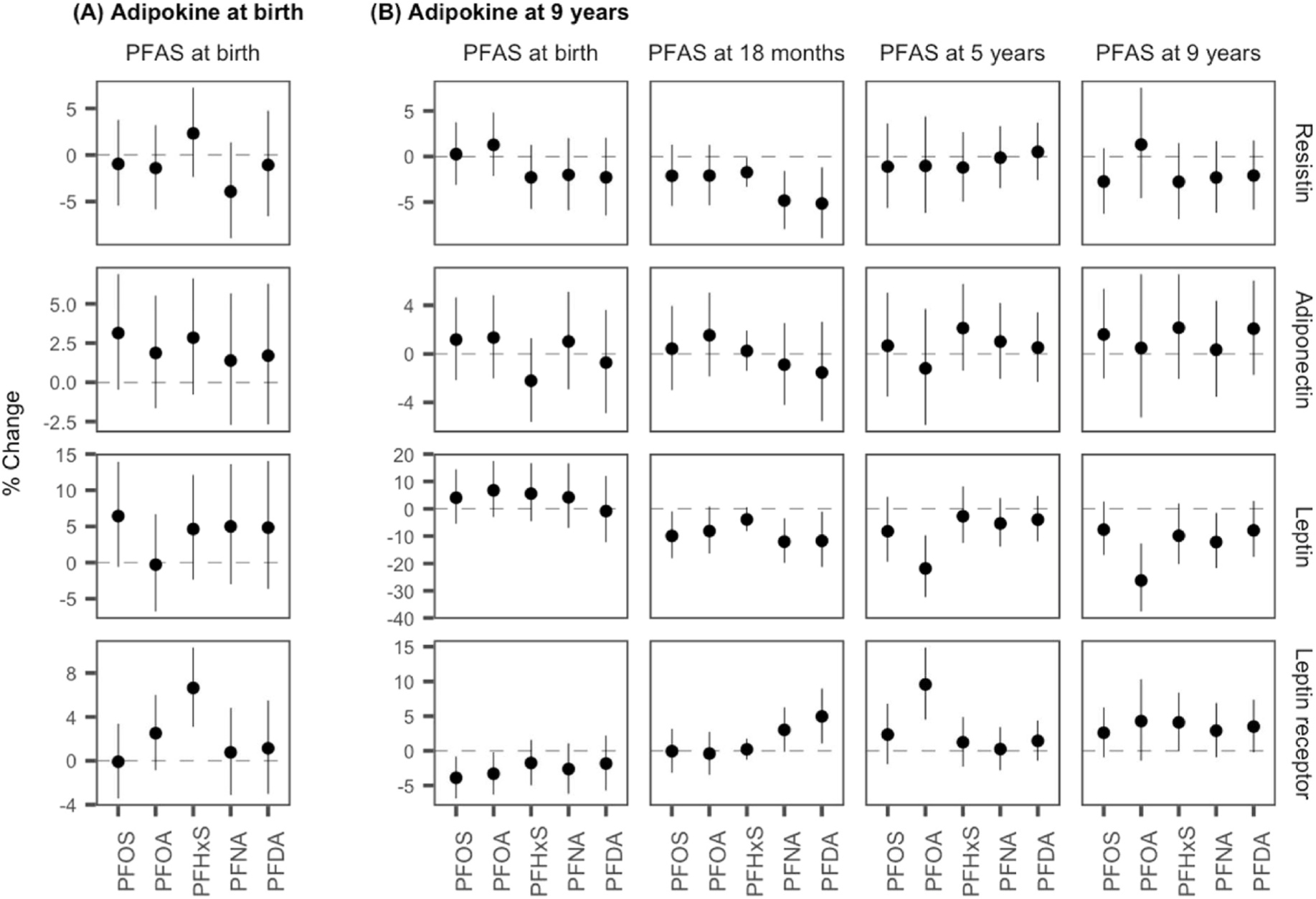
Percent change of serum-adipokine hormone concentrations at birth (A) and age 9 years (B) per doubling of the serum-PFAS concentrations at birth, 18 months, and 5 and 9 years in the overall study population.

The results from the single-PFAS linear regression models in the overall study population are shown in Fig. 1 and Table S1–S5. For serum-adipokine concentrations at birth, only one significant association was observed, i.e., between PFHxS and the leptin receptor; a doubling of PFHxS at birth was associated with a 6.65% (95% CI: 3.10, 10.32) increase in leptin receptor.

For serum-adipokine concentrations at age 9 years, only a few associations were observed with serum-PFAS concentrations at birth; a doubling of PFOA and PFOS was associated with a 3.89% (95% CI: −6.86, −0.93) and a 3.30% (95% CI: −6.30, −0.20) decrease in leptin receptor, respectively. More associations with adipokine concentrations at age 9 years were observed in relation to serum-PFAS concentrations at 18 months. A doubling of PFOS was associated with a 9.91% (95% CI: −18.00, −1.02) decrease in leptin, and a doubling of PFHxS was associated with a 1.73% (95% CI: −3.33, −0.12) decrease in resistin. In addition, both PFNA and PFDA were associated with decreased resistin and leptin as well as increased leptin receptor. For serum-PFAS concentrations at 5 years, PFOA was the only PFAS showing significant associations with adipokine concentrations at age 9 years; a doubling of PFOA was associated with a 21.81% (95% CI: −32.28, −9.73) decrease in leptin and a 9.57% (95% CI: 4.50, 14.88) increase in leptin receptor. Serum-PFOA concentrations at 9 years also showed strong associations with leptin at that age; a doubling of PFOA was associated with a 26.22% (95% CI: −37.64, −12.70) decrease in leptin. Further, a doubling of PFNA at age 9 years was associated with a 12.17% (95% CI: −21.65, −1.54) decrease in leptin. Lep/LepR ratio and Adpn/Lep ratio also showed significant associations with PFASs at 18 months, and 5 and 9 years, while these associations may simply be reflections of the changes in leptin since both ratios were highly correlated with leptin (Spearman’s rho = 0.98 for Lep/LepR ratio; Spearman’s rho = −0.95 for Adpn/Lep ratio).

In analyses where an interaction term between PFAS and sex was included in the model, almost all models found no significant modification effect of sex on the associations of PFAS concentrations with adipokine concentrations at birth and age 9 years (Figure S5 and Table S1–S5). While serum PFHxS concentrations at birth were associated with increased leptin receptor at birth among females, it was null for males (P_interaction_ = 0.19). Further, the effects of PFAS concentrations at 18 months and 5 years on serum-resistin concentration at 9 years appeared to be sex-specific. For example, although the confidence interval contains the null, an inverse trend was observed between PFOS at 5 years and resistin at 9 years among females, while a positive trend was observed in males (P_interaction_ = 0.04).

### 3.2. Bayesian kernel machine regression (BKMR)

**Fig. 2.**
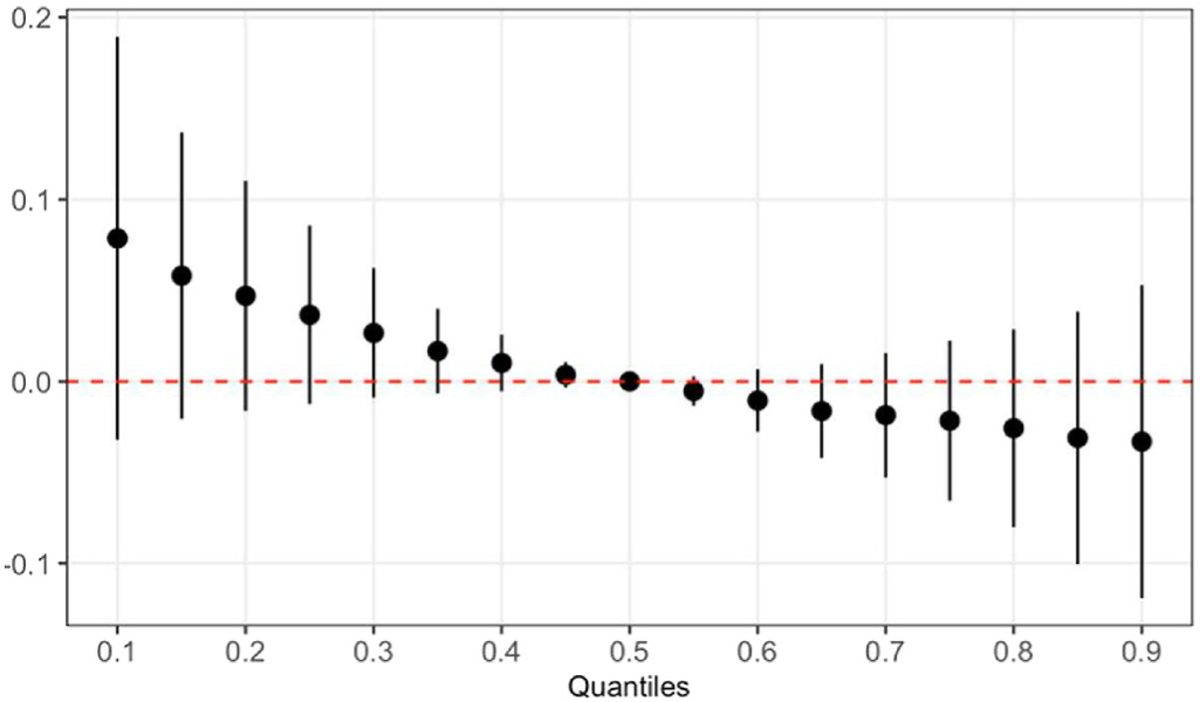
The effect of overall PFAS mixtures at 18 months on resistin at 9 years estimated by Bayesian kernel machine regression models in the overall study population.

**Fig. 3.**
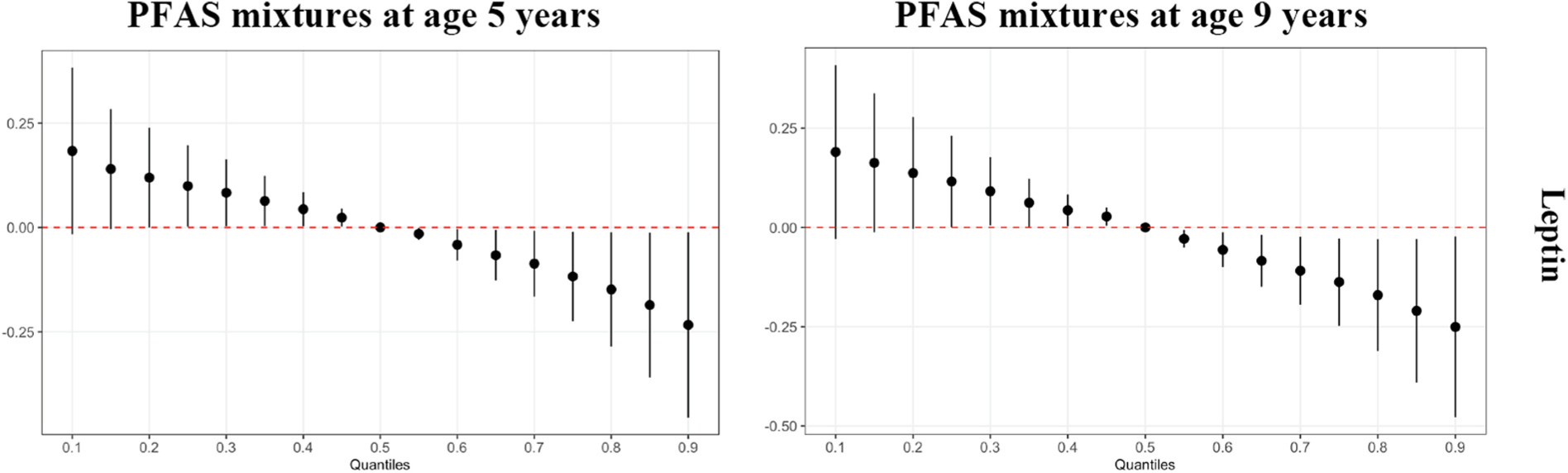
The effect of overall PFAS mixtures at 5 and 9 years on leptin at 9 years estimated by Bayesian kernel machine regression models in the overall study population.

**Fig. 4.**
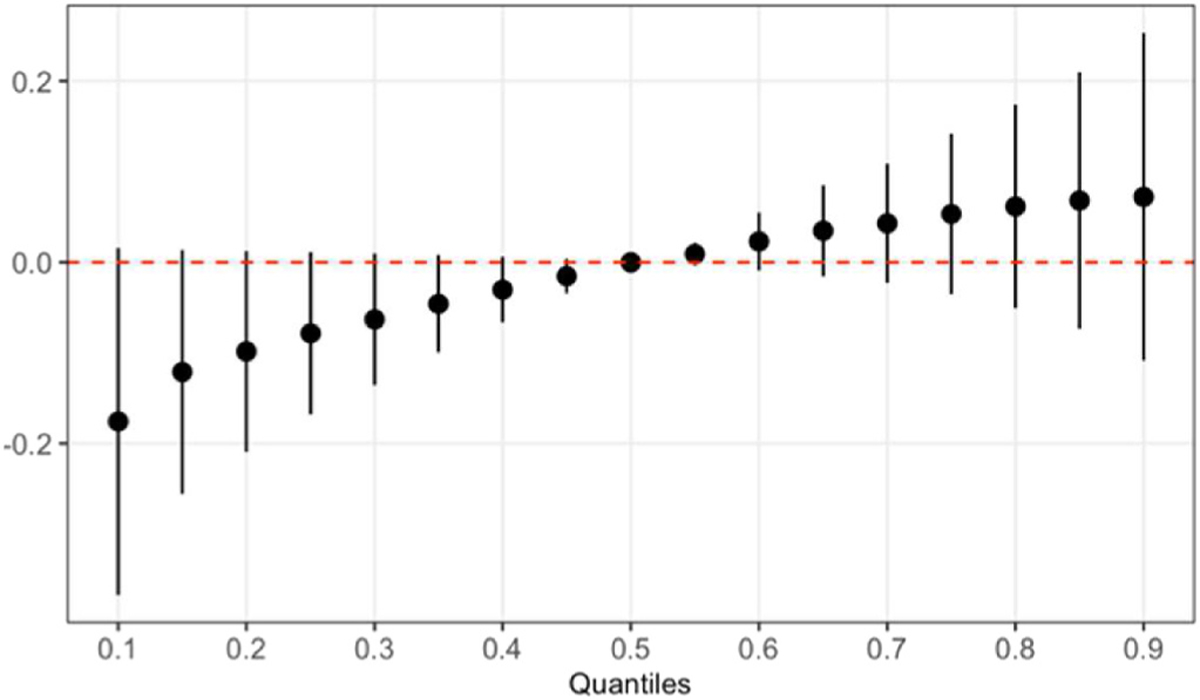
The effect of overall PFAS mixtures at 5 years on leptin receptor at 9 years estimated by Bayesian kernel machine regression models in the overall study population.

In our BKMR analyses, we found no significant association between the overall PFAS mixture at birth and the concomitant adipokine concentrations. For age 9 year adipokines, we observed an inverse trend between resistin and the PFAS mixture at 18 months, although the confidence interval contained the null (Fig. 2). PFNA was identified as the most important PFAS at 18 months in the mixture model with decreased resistin based on a PIP of 0.70 and its dose-response function (Table S7 and Figure S6). In addition, the overall PFAS mixtures at 5 and 9 years were significantly associated with decreased leptin at 9 years (Fig. 3). Based on PIPs, PFOA was identified (i.e., 0.90 for 5-year PFAS and 0.93 for 9-year PFAS) and the dose response function to be the only and primary PFAS driving these associations (Table S7 and Figure S7). Furthermore, a positive trend was observed between the overall PFAS mixtures at 5 years and leptin receptor at 9 years (Fig. 4), and PFOA was also identified as the primary PFAS driving this trend based on a PIP of 0.88 and its dose response function (Table S7 and Figure S8). We observed no significant association between the overall PFAS mixture at birth and adipokine concentrations at 9 years (data not shown). All single-PFAS associations with adipokine concentrations, adjusting for all other PFASs fixed at their median value, appeared linear and consistent with the findings from our linear regression models (data not shown). No significant pairwise interactions between PFASs and adipokine concentrations were observed (data not shown).

In BKMR analyses stratified by sex, we observed similar trends for the associations of the overall PFAS mixtures with adipokine hormones for males and females, with the same directionality as in the overall study population. However, none of these trends was significant, possibly due to smaller sample sizes (data not shown).

### 3.3. Sensitivity analysis

In the GEE models where we jointly estimated the associations of repeated serum-PFAS concentrations measured at birth, 18 months, and 5 and 9 years with serum-adipokine concentrations at 9 years, we observed the same significant PFAS-adipokine associations as in the primary analyses using linear regression models (Figure S9 and S10), although the effect estimates were generally smaller with narrower confidence intervals. For example, PFOA at 5 years were strongly associated with decreased leptin and increased leptin receptor at age 9 years. Also, like linear regression models, only a few significant modification effects of sex were observed.

In sensitivity analyses where duration of exclusive breastfeeding, maternal whale consumption during pregnancy, and child whale consumption at 9 years were included as covariates in separate regression models, the association estimates of serum-PFAS concentrations on adipokine concentrations were of a similar magnitude and in the same direction, thus suggesting no important confounding by these variables (Figure S11–S13).

## 4. Discussion

In this study, we evaluated the effects of prenatal and childhood exposures to PFASs on adipokine hormones measured at birth and age 9 years in Faroese children. We observed associations of PFASs at 18 months and 5 and 9 years with increased leptin receptor and decreased resistin and leptin at 9 years. Further, inverse associations were found for PFASs at birth and the leptin receptor at 9 years. These findings were confirmed by the BKMR analyses that showed significant associations of the overall PFAS mixtures at 18 months and 5 and 9 years with adipokine concentrations at 9 years.

The present study follows a previous report on PFAS exposure and adipokine hormones among children in the Faroe Islands. The first study included 80 mother-child pairs from an older cohort and evaluated the associations of the same five PFASs in maternal serum and in child serum from ages 5, 7, and 13 years with serum concentrations of resistin, adiponectin, and leptin in cord blood and at the same ages (Shelly et al., 2019). Unlike the present study, most of the significant associations were sex-specific. Thus, inverse associations of maternal serum concentrations of PFOS, PFNA, and PFDA with resistin at birth and 5 years were observed among males only, whereas the present study found inverse associations of PFNA and PFDA at 18 months with resistin at 9 years in the overall study population. Further, the previous study reported inverse associations of maternal serum concentrations of PFOS, PFHxS, PFNA, and PFDA with cord blood adiponectin only among females, while we found no evidence of the association between serum-PFAS concentrations and adiponectin. Lastly, both studies observed inverse associations between more recent childhood serum-PFAS concentrations and leptin, but the associations were primarily with PFOS among females in the first study, while the associations observed in the present study was primarily for PFOA. These findings may not be inconsistent, as exposures to legacy PFASs (i.e., PFOA, PFOS, and PFHxS) in the older cohort were much higher compared with the exposure range reported in the present cohort, where exposures to PFNA and PFDA appeared to be slightly elevated.

In the present study, we observed a positive association between PFHxS and the leptin receptor, both measured at birth, while no evidence was found for the other adipokine measures. Like leptin, the leptin receptor is one of the key regulators of energy homeostasis (Gruzdeva et al., 2019); however, to our knowledge, no studies so far have explored its possible association with PFAS exposure. Previous studies on adipokine hormones at birth primarily focused on leptin and adiponectin, and somewhat mixed results have been reported. In the HOME study of 230 mother-infant pairs, no associations were seen between the 2nd trimester serum-PFAS concentrations (i.e., PFOA, PFOS, PFHxS, and PFNA) and cord blood leptin and adiponectin (Buck et al., 2018). When stratifying by sex, PFNA was associated with increased leptin in males but decreased leptin in females. Further, the MIREC study of 1705 mother-infant pairs reported no associations of the 1st trimester plasma PFOA, PFOS, and PFHxS with cord blood leptin and adiponectin (Ashley-Martin et al., 2017). The Hokkaido study of 168 mother-infant pairs observed a positive association between maternal PFOS and cord blood adiponectin (Minatoya et al., 2017). These somewhat inconsistent findings across studies may well be due to different study windows and PFAS exposure profiles.

Only few studies have examined the association between PFAS exposure and childhood adipokine hormones, and the present findings differ somewhat from previous studies. We found that serum-PFAS concentrations at 18 months (i.e., PFOS, PFNA, and PFDA), 5 (i.e., PFOA) and 9 years (i.e., PFOA and PFNA) were associated with decreased leptin at 9 years. However, a cross-sectional analysis of 501 children aged 9 years from the European Youth Heart Study observed an inverse association only between PFHxS and leptin at 9 years (Domazet et al., 2020). Further, in Project Viva with 665 mother-child pairs, no associations of maternal PFAS concentrations or childhood PFAS concentrations were found with leptin during mid-childhood (Fleisch et al., 2017).

This study is the first to investigate the associations of PFAS concentrations with childhood leptin receptor concentrations. We found that serum-PFAS concentration at 18 months (i.e., PFDA) and 5 years (i.e., PFOA) were associated with increased leptin receptor at age 9 years. The leptin receptor is a transmembrane protein that can transduce the brain leptin signaling system, and previous studies have linked lower leptin receptor concentrations to obesity (Sudhakar et al., 2018). Given that PFAS exposure may be associated with altered leptin and leptin receptor concentration in children, and these alterations may be further linked to metabolic health later in life, childhood adipokine hormone concentrations should be considered for inclusion in future studies of PFAS effects on metabolic functions.

BKMR analyses evaluating PFAS mixtures supported the associations observed in the linear regression models and did not detect any nonlinear associations or strong interaction effects. Results from the BKMR models confirmed an association between overall PFAS concentrations at 18 months and resistin at age 9, with PFNA contributing the most. In regard to PFAS concentrations at 5 and 9 years, the association with leptin at age 9 was the strongest, and PFOA appeared to be the primary contributor. These results highlight how BKMR can be applied when PFAS concentrations are highly correlated to tease out the contributions of each individual PFAS on the outcomes of interest.

This study leveraged a large prospective birth cohort to investigate the associations between serum-PFAS concentrations measured at different time points and adipokine concentrations at birth and age 9 years, providing a contribution to our current understanding of the potential biological pathway connecting PFAS and metabolic health. However, there are important limitations to consider. First, findings from the present study may be subject to potential multiple testing issue that could result in a Type I error, although our use of the BKMR suggested that the associations identified were robust. Second, adipokine hormones fluctuate throughout the day based on the peripheral circadian clock (Gomez-Abellan et al., 2010; Oliver et al., 2006), which may result in potential imprecision of our outcome variables. Third, we measured total adiponectin, rather than high-molecular-weight (HMW) adiponectin, which by some is considered the biologically most active form of adiponectin and possibly a better indicator of glucose homeostasis and insulin sensitivity (Wang et al., 2008). Still, the predictive validity of adiponectin analyses is likely to be dependent on the assay used, and our adiponectin analyses showed excellent accuracy. Further, residual confounding may be present due to unmeasured variables, such as details on diet and physical activity (e.g., energy expenditure) and other behavioral or socio-economics status indicators. Finally, findings from the present study may not be generalizable to populations with different PFAS exposure profiles and backgrounds.

## 5. Conclusions

In this prospective cohort of Faroese mother-child pairs, we found that serum-PFHxS at birth was associated with increased leptin receptor at birth, and that childhood concentrations of PFASs were associated with decreased resistin and leptin and increased leptin receptor at age 9 years. Further, an overall mixture effect of childhood PFAS exposures on leptin at age 9 years was suggested by the BKMR analyses. Limited evidence was observed for potential sex-specific association between serum-PFAS concentrations and adipokine hormones. Given the apparent impact of PFAS exposure on metabolic health outcomes and the associations of adipokine hormones with both PFAS exposure and metabolic health, future studies are warranted to explore causal links and to identify the mechanism by which adipokine hormones may serve as mediators between PFAS exposure and metabolic health. Ultimately, serum-adipokine concentrations may conceivably serve as relevant biomarkers that can help identify children at risk and inform public health interventions.

## Supplementary Material

1

## Figures and Tables

**Table 1:** Characteristics of participants included in analysis of PFAS concentrations and adipokine hormones at birth.

Characteristics	Total cohort (n = 463)	Male (n = 241; 52%)	Female (n = 222; 48%)	P-value

Maternal age, mean ± SD	29.75 ± 5.56	30.26 ± 5.54	29.20 ± 5.55	0.07
Maternal education, n (%)				0.16
Low	153 (33.0)	71 (29.5)	82 (36.9)	
Medium	120 (25.9)	62 (25.7)	58 (26.1)	
High	190 (41.0)	108 (44.8)	82 (36.9)	
Maternal smoking, n (%)				0.20
No	391 (84.4)	206 (85.5)	185 (83.3)	
1–5 cigarettes per day	38 (8.2)	22 (9.1)	16 (7.2)	
>5 cigarettes per day	34 (7.3)	13 (5.4)	21 (9.5)	
Parity, n (%)				0.04
Primiparous	136 (29.4)	60 (24.9)	76 (34.2)	
Multiparous	327 (70.6)	181 (75.1)	146 (65.8)	
Pre-pregnancy BMI (kg/m^2^), mean ± SD	24.35 ± 4.31	24.69 ± 4.53	24.98 ± 4.04	0.07
Maternal whale consumption, n (%)^1^				0.10
Yes	93 (20.4)	56 (23.4)	37 (17.0)	
No	364 (79.6)	183 (76.6)	181 (83.0)	
Exclusive breastfeeding (month), mean ± SD^2^	4.50 (2.06)	4.37 (2.13)	4.65 (1.98)	0.19

a Chi-square test for maternal education, maternal smoking, and parity, and Wilcoxon rank-sum test for maternal age and pre-pregnancy BMI.

b SD: standard deviation; BMI: body mass index.

1 Among 457 mother-child pairs (239 males and 218 females).

2 Among 428 mother-child pairs (227 males and 201 females).

**Table 2:** Median [interquartile range] of adipokine hormones at birth and age 9 years.

Adipokine hormone	Total cohort	Male	Female	P-value^a^

Resistin (ng/ml)				
At birth	16.00 [11.45–21.80]	14.40 [10.8–20.50]	17.15 [13.00–24.08]	<0.001
At 9 years	3.10 [2.43–3.84]	2.90 [2.36–3.48]	3.26 [2.61–4.06]	<0.001
Adiponectin (ng/ml)				
At birth	31.08 [24.12–39.78]	30.00 [23.40–39.72]	32.10 [24.83–39.90]	0.17
At 9 years	12.48 [10.32–15.11]	12.00 [9.95–14.57]	13.44 [10.74–15.54]	0.005
Leptin (ng/ml)				
At birth	7.71 [4.37–12.85]	6.17 [3.57–10.00]	9.92 [5.73–15.78]	<0.001
At 9 years	2.66 [1.30–5.17]	1.72 [0.71–4.02]	3.98 [2.00–6.35]	<0.001
Leptin receptor (ng/ml)				
At birth	12.72 [10.17, 16.50]	13.29 [10.71–17.52]	11.87 [9.37–15.68]	0.002
At 9 years	29.90 [23.72–38.10]	32.10 [26.87–40.58]	26.74 [21.98–33.53]	<0.001

a Wilcoxon rank-sum test.
