# Supplementary material for: Early-life exposure to perfluoroalkyl substances in relation to serum adipokines in a longitudinal birth cohort
Source: Environ Res. Author manuscript; Available in PMC 2024 Mar 11. (PMC10926841; doi:10.1016/j.envres.2021.111905)
Supplement: 1 [file NIHMS1960377-supplement-1.pdf]

Supplemental Materials

Early-life exposure to perfluoroalkyl substances in relation to serum adipokines in a longitudinal birth cohort

Yu-Hsuan Shih,[1] Annelise J. Blomberg,[1,2] Louise Helskov Jørgensen,[3] Pál Weihe,[4,5] Philippe Grandjean[1,6*]

[1] Department of Environmental Health, Harvard T.H. Chan School of Public Health, Boston, MA 02115
[2] Division of Occupational and Environmental Medicine, Lund University, Lund, Sweden
[3] Department of Clinical Biochemistry and Pharmacology, Odense University Hospital and Institute of Clinical Research, University of Southern Denmark, Odense Denmark
[4] Department of Occupational Medicine and Public Health, Faroese Hospital System, Torshavn The Faroe Islands
[5] Center of Health Science, University of the Faroe Islands, Torshavn The Faroe Islands
[6] Department of Environmental Medicine, University of Southern Denmark, Odense, Denmark

Supplemental Figures

Figure S1. Directed acyclic graph of the hypothesized causal pathway between child serum-PFAS concentrations at birth, 18 months, and 5 years and child serum-adipokine concentrations at birth and 9 years …………………………………………………….. 3

Figure S2. Directed acyclic graph of the hypothesized causal pathway between child serum-PFAS concentrations at 9 years and child serum-adipokine concentrations at 9 years …….. 4

Figure S3. Distribution of serum-PFAS concentrations at birth, 18 months, and 5 and 9 years, overall and by sex ………………………………………………………………….... 5

Figure S4. Spearman correlation coefficients between serum-PFAS concentrations measured at birth, 18 months, and 5 and 9 years ………………..…………………………………….. 6

Figure S5. Sex-specific percent change of serum-adipokine hormone concentrations at birth and age 9 years per doubling of the serum-PFAS concentrations at birth, 18 months, and 5 and 9 years ……………………………………………………………………………….. 7

Figure S6. Dose response function between each serum-PFAS concentration at 18 month and resistin at 9 years ……………………………………………………...…………….... 8

Figure S7. Dose response function between each serum-PFAS concentration at 5 (A) and 9 years (B) and leptin at 9 years ……………………………………...………………….... 9

Figure S8. Dose response function between each serum-PFAS concentration at 5 years and leptin receptor at 9 years ………………………………………………………………… 10

Figure S9. Comparison between results using generalized estimating equations to primary results using linear regression models in the overall study population. Results are presented as the percent change of serum-adipokine hormone concentrations at age 9 years per doubling of the serum-PFAS concentrations at birth, 18 months, and 5 and 9 years …………………… 11

Figure S10. Comparison between sex-specific results using generalized estimating equations to primary results using linear regression models. Results are presented as the percent change of serum-adipokine hormone concentrations at age 9 years per doubling of the serum-PFAS concentrations at birth, 18 months, and 5 and 9 years ……………………………………… 12

Figure S11. Comparison of effect estimates from the primary models and models additionally including duration of exclusive breastfeeding for the associations of serum-PFAS concentrations at 18 months and 5 years with serum-adipokine hormone concentrations at age 9 years …..…………………………………………………………………………………... 13

Figure S12. Comparison of effect estimates from the primary models and models additionally including maternal whale consumption during pregnancy for the associations of serum-PFAS concentrations at birth, 18 months, and 5 years with serum-adipokine hormone concentrations at birth and age 9 years ………..…………………………………………………………….. 14

Figure S13. Comparison of effect estimates from the primary models and models additionally including child whale consumption at 9 years for the associations of serum-PFAS concentrations at 9 years with serum-adipokine hormone concentrations at age 9 years …... 15

Supplemental Tables

Table S1. Percent change of the serum-adipokine concentrations at birth per doubling of the serum-PFAS concentrations at birth, overall (n = 463) and by sex (male = 241; female = 222) ………………………………………………………………………………………. 16

Table S2. Percent change of the serum resistin concentrations at age 9 years per doubling of the serum-PFAS concentrations at birth, ages 18 months, and 5 and 9 years, overall and by sex ……………………………………………………………………………………... 17

Table S3. Percent change of the serum adiponectin concentrations at age 9 years per doubling of the serum-PFAS concentrations at birth, ages 18 months, and 5 and 9 years, overall and by sex …………………………………………………………………………………….. 18

Table S4. Percent change of the serum leptin concentrations at age 9 years per doubling of the serum-PFAS concentrations at birth, ages 18 months, and 5 and 9 years, overall and by sex ……………………...…………………………………………………………………… 19

Table S5. Percent change of the serum leptin receptor concentrations at age 9 years per doubling of the serum-PFAS concentrations at birth, ages 18 months, and 5 and 9 years, overall and by sex ………………………………………………………………………...... 20

Table S6. Posterior inclusion probabilities (PIPs) of five PFASs measured at birth in relation to the serum-adipokine concentrations at birth in the overall population ……….……..…... 21

Table S7. Posterior inclusion probabilities (PIPs) of five PFASs measured at birth, 18 months, and 5 and 9 years in relation to the serum-adipokine concentrations at 9 years in the overall population ………………………………………………………………………………... 22

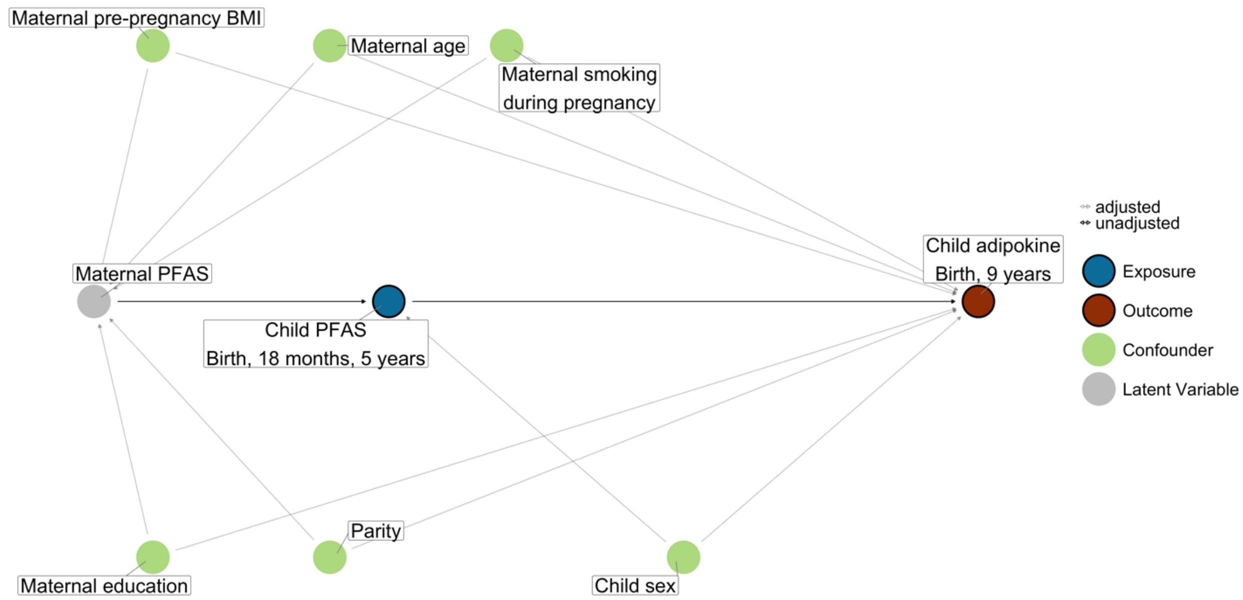

Figure S1. Directed acyclic graph of the hypothesized causal pathway between child serum-PFAS concentrations at birth, 18 months, and 5 years and child serum-adipokine concentrations at birth and 9 years.

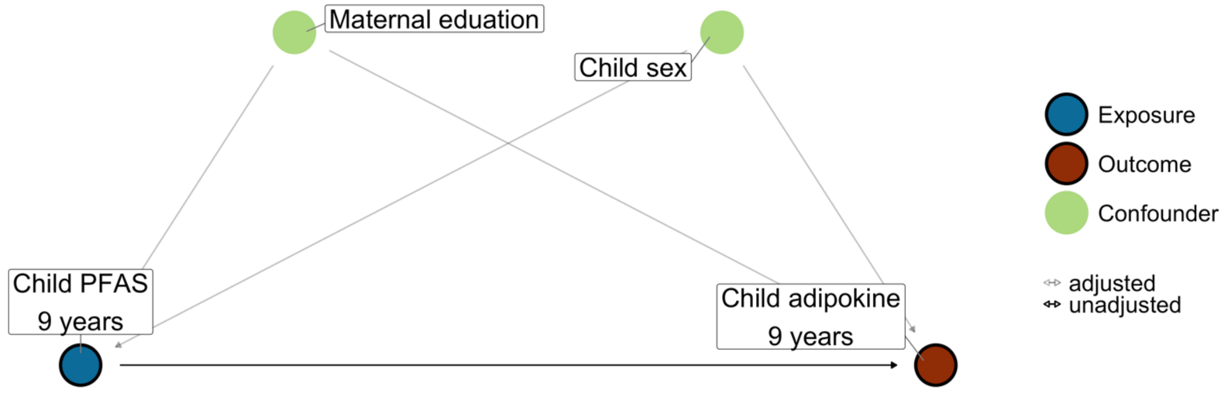

Figure S2. Directed acyclic graph of the hypothesized causal pathway between child serum-PFAS concentrations at 9 years and child serum-adipokine concentrations at 9 years.

(A) All

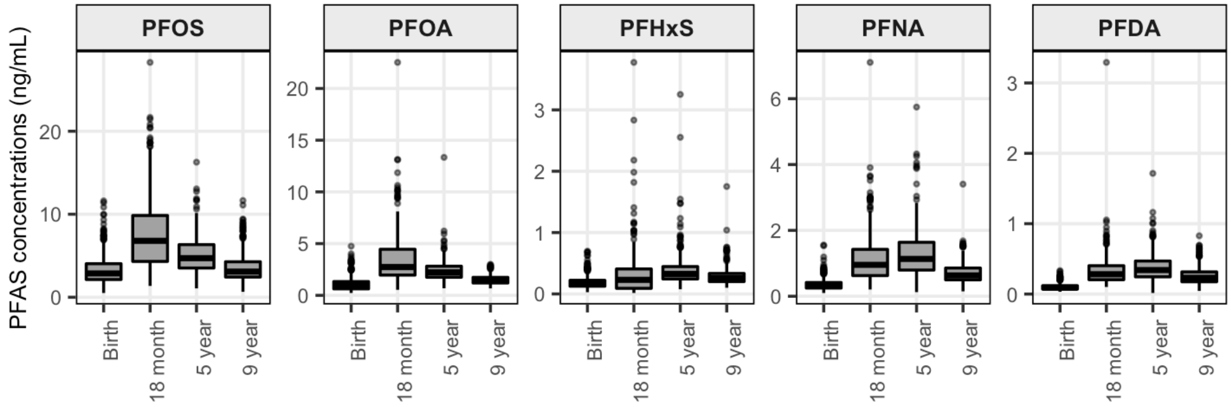

(B) By sex

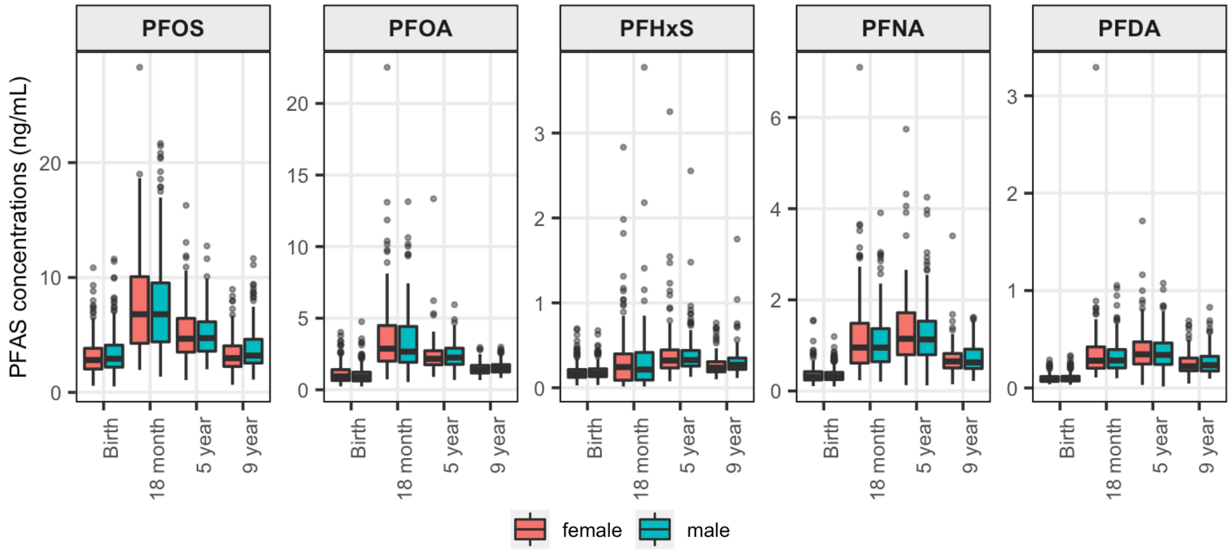

Figure S3. Distribution of serum-PFAS concentrations at birth, 18 months, and 5 and 9 years, overall and by sex.

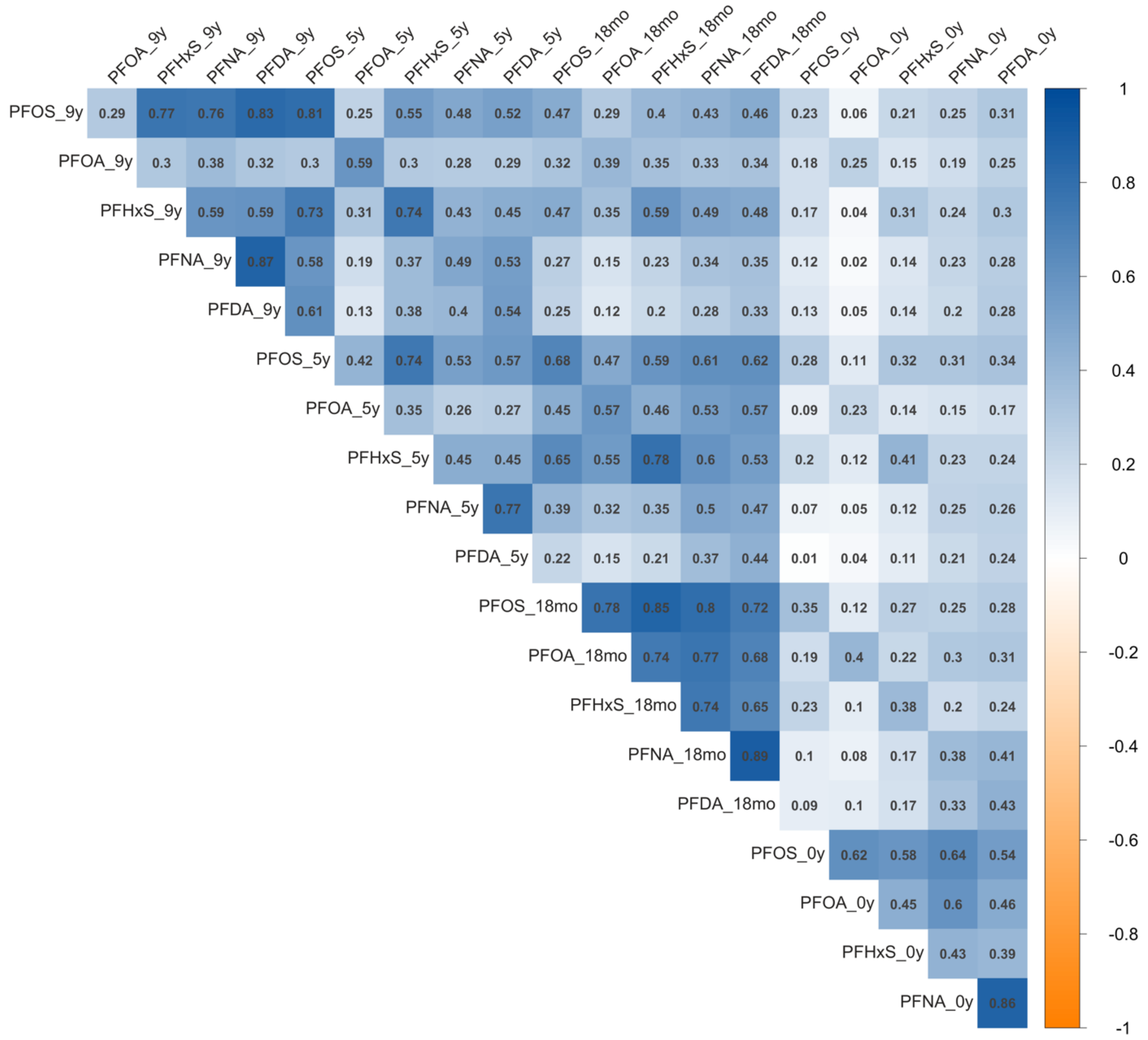

Figure S4. Spearman correlation coefficients between serum-PFAS concentrations measured at birth, 18 months, and 5 and 9 years.

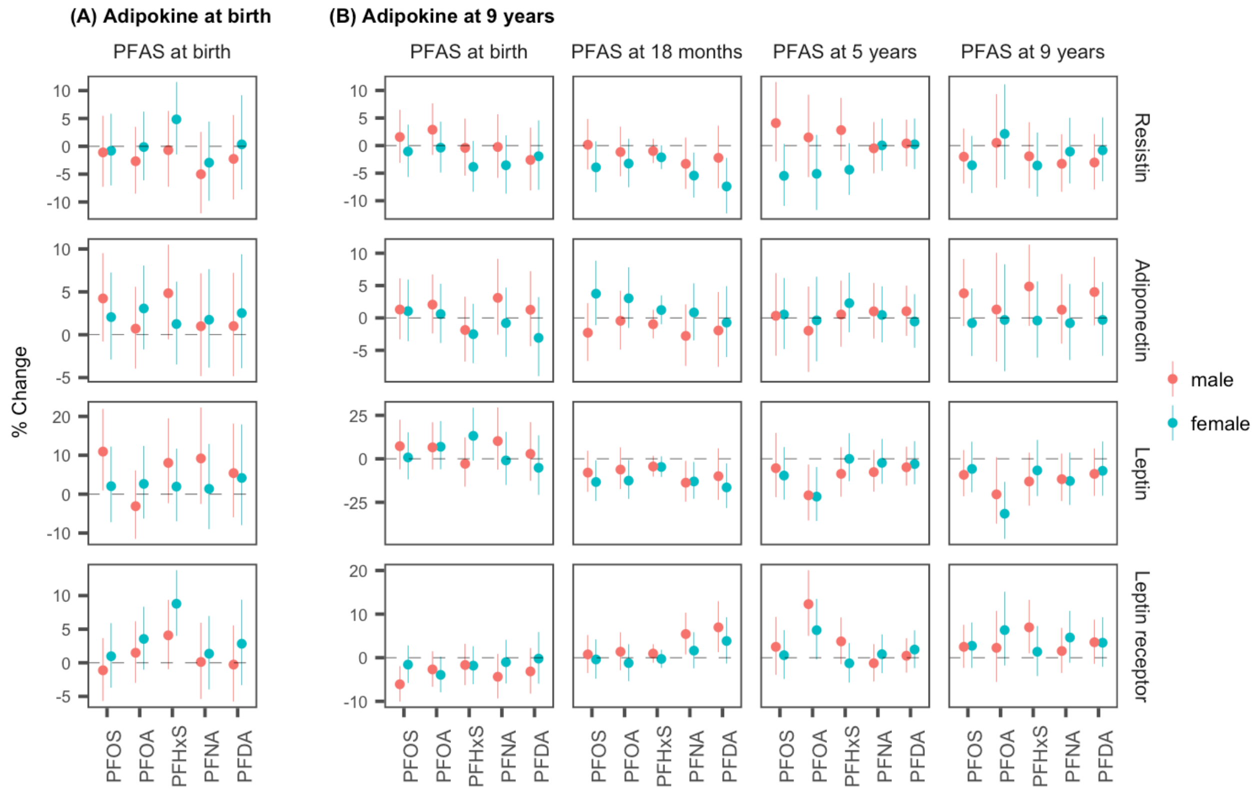

Figure S5. Sex-specific percent change of serum-adipokine hormone concentrations at birth and age 9 years per doubling of the serum-PFAS concentrations at birth, 18 months, and 5 and 9 years.

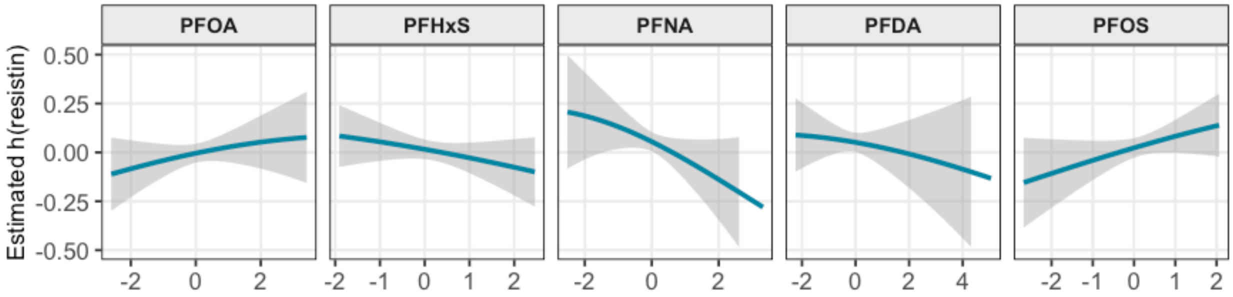

Figure S6. Dose response function between each serum-PFAS concentration at 18 month and resistin at 9 years.

(A) PFAS concentrations at age 5 years

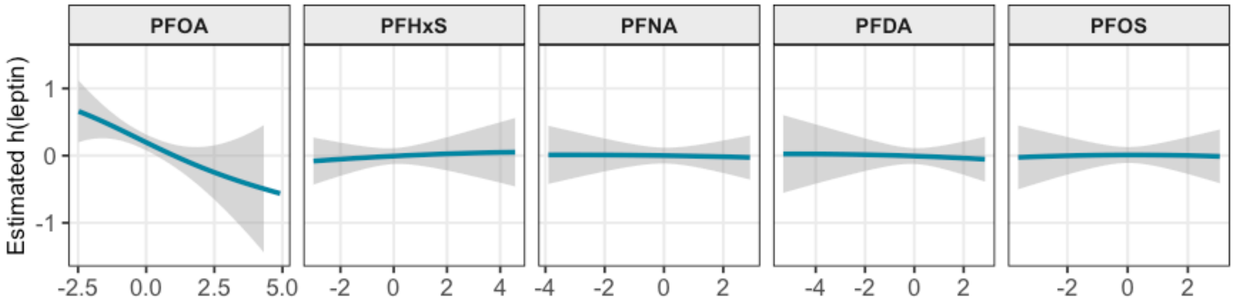

(B) PFAS concentrations at age 9 years

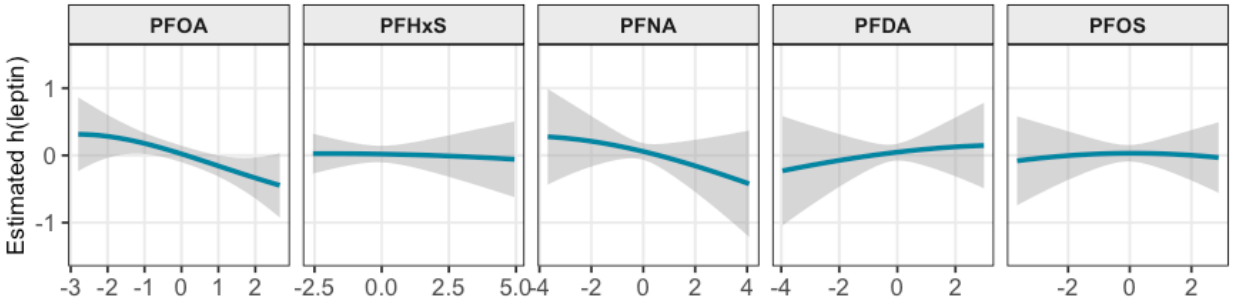

Figure S7. Dose response function between each serum-PFAS concentration at 5 (A) and 9 years (B) and leptin at 9 years.

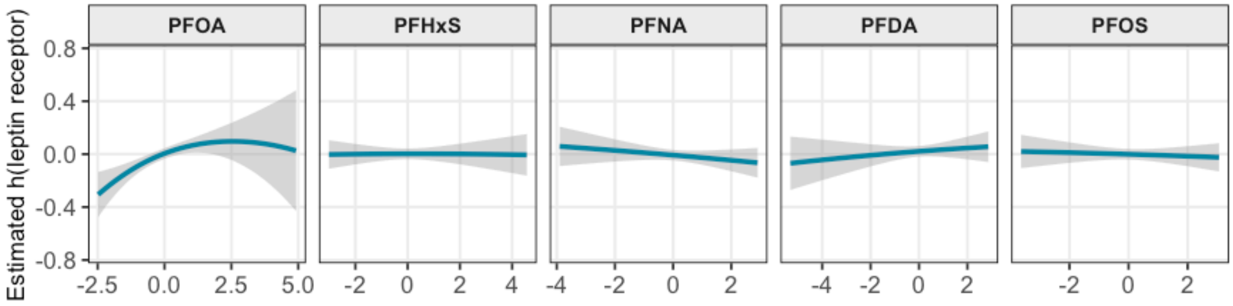

Figure S8. Dose response function between each serum-PFAS concentration at 5 years and leptin receptor at 9 years.

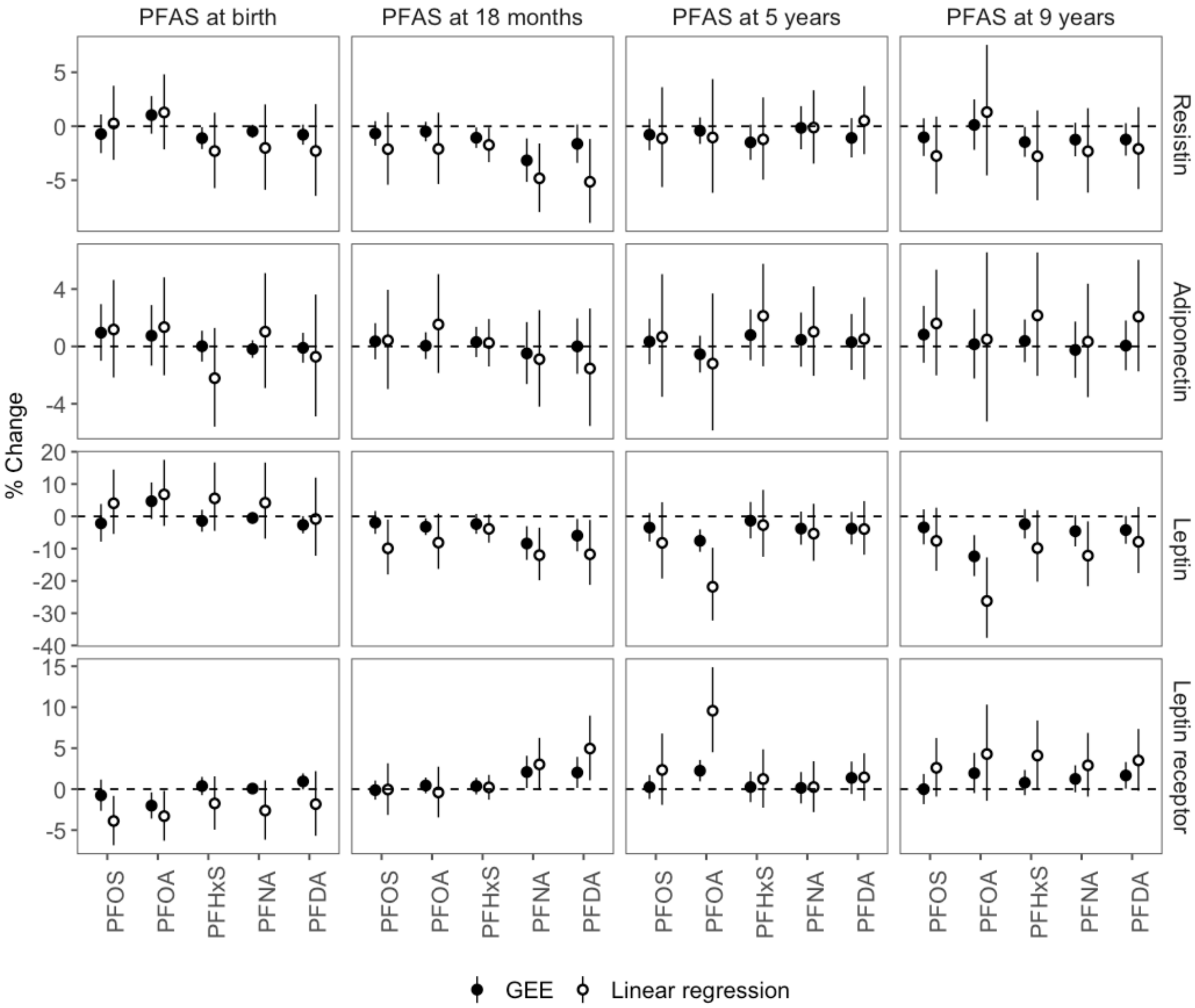

Figure S9. Comparison between results using generalized estimating equations to primary results using linear regression models in the overall study population. Results are presented as the percent change of serum-adipokine hormone concentrations at age 9 years per doubling of the serum-PFAS concentrations at birth, 18 months, and 5 and 9 years.

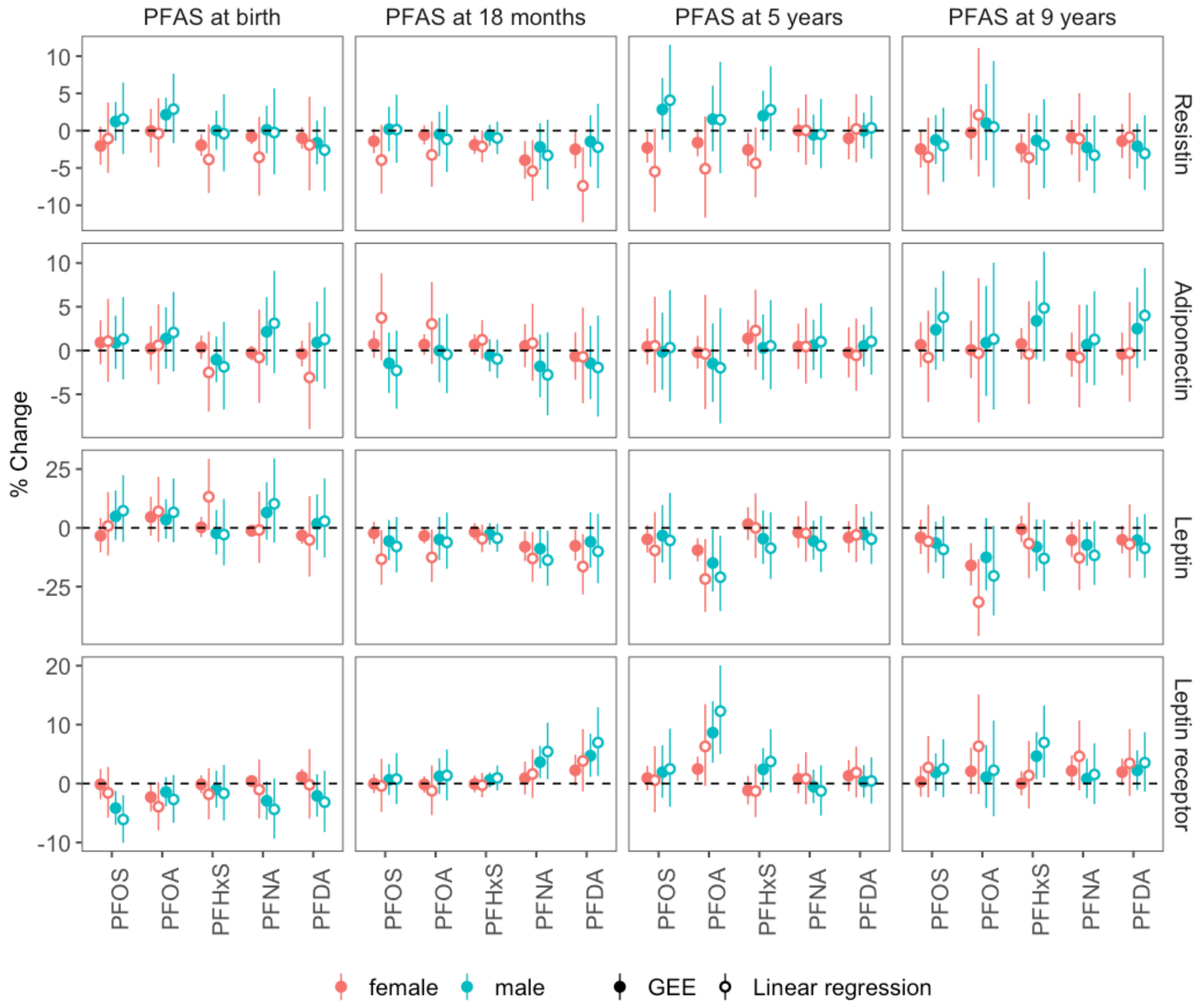

Figure S10. Comparison between sex-specific results using generalized estimating equations to primary results using linear regression models. Results are presented as the percent change of serum-adipokine hormone concentrations at age 9 years per doubling of the serum-PFAS concentrations at birth, 18 months, and 5 and 9 years.

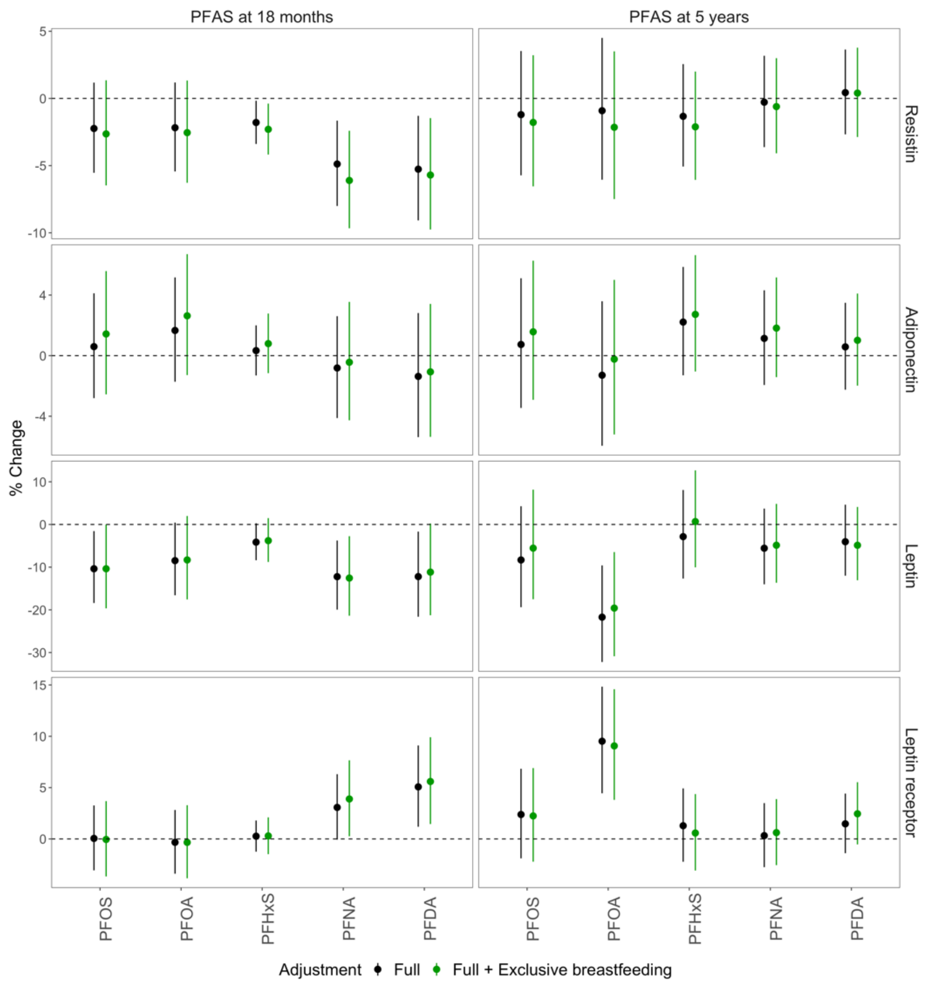

Figure S11. Comparison of effect estimates from the primary models and models additionally including duration of exclusive breastfeeding for the associations of serum-PFAS concentrations at 18 months and 5 years with serum-adipokine hormone concentrations at age 9 years.

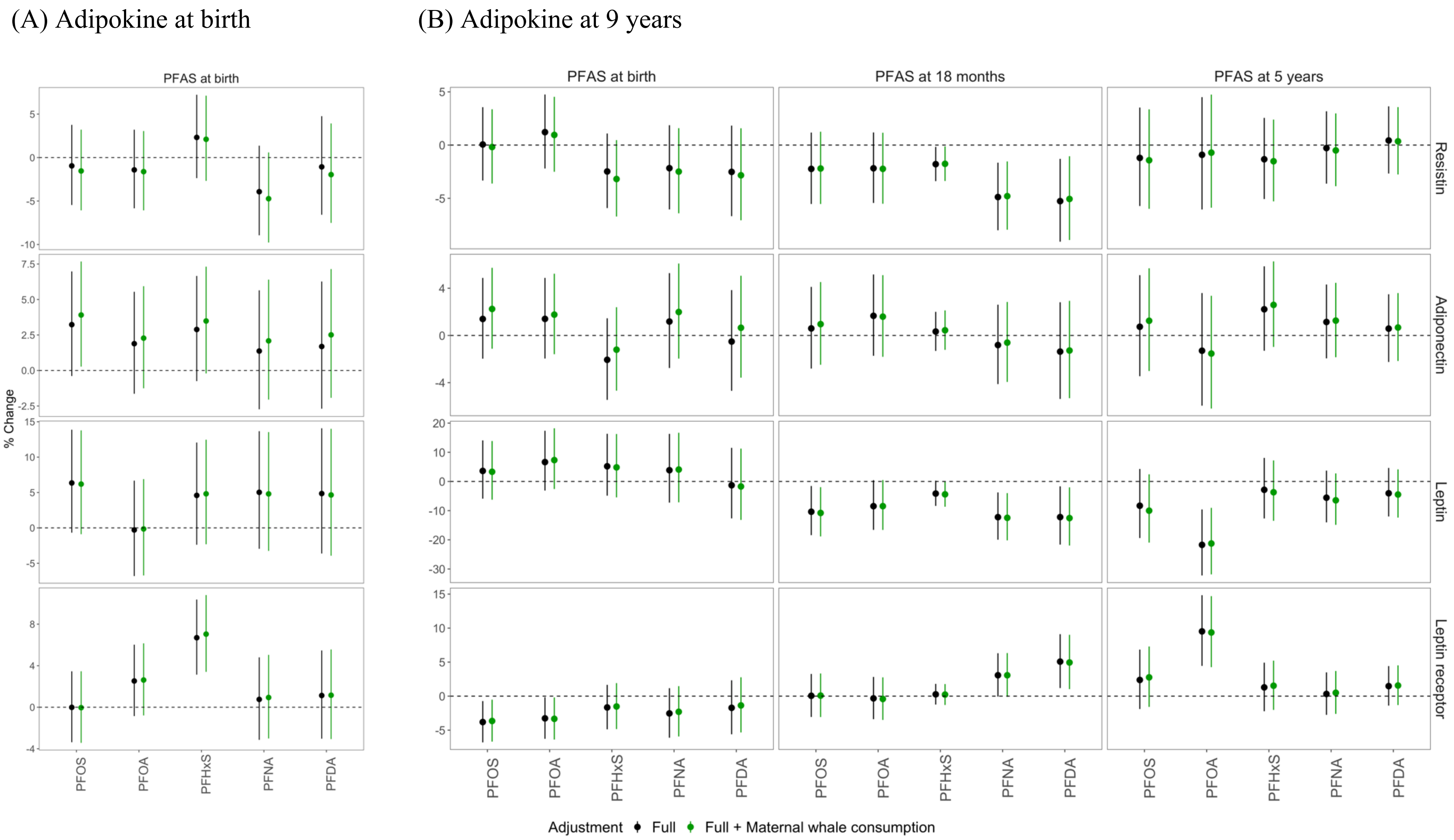

Figure S12. Comparison of effect estimates from the primary models and models additionally including maternal whale consumption during pregnancy for the associations of serum-PFAS concentrations at birth, 18 months, and 5 years with serum-adipokine hormone concentrations at birth and age 9 years.

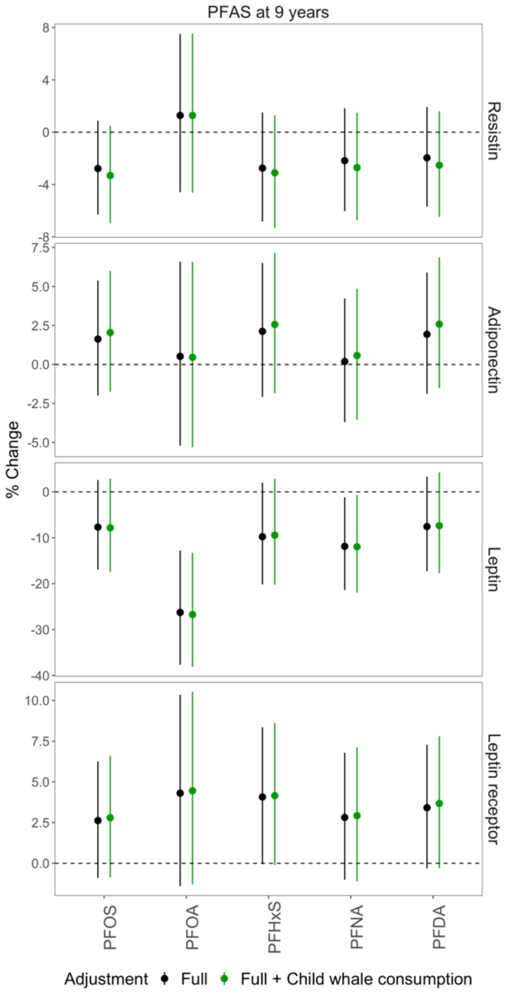

Figure S13. Comparison of effect estimates from the primary models and models additionally including child whale consumption at 9 years for the associations of serum-PFAS concentrations at 9 years with serum-adipokine hormone concentrations at age 9 years.

Table S1. Percent change of the serum-adipokine concentrations at birth per doubling of the serum-PFAS concentrations at birth, overall (n = 463) and by sex (male = 241; female = 222).

Adipokine	Total cohort		Male		Female		P for interaction
	% change	95% CI	% change	95% CI	% change	95% CI	
Resistin							
PFOS	-0.95	-5.44, 3.76	-1.10	-7.26, 5.48	-0.80	-7.02, 5.84	0.95
PFOA	-1.42	-5.83, 3.2	-2.68	-8.48, 3.49	-0.13	-6.1, 6.22	0.54
PFHxS	2.32	-2.36, 7.22	-0.69	-7.25, 6.33	4.84	-1.46, 11.54	0.24
PFNA	-3.93	-8.94, 1.36	-5.01	-12.04, 2.58	-2.94	-9.78, 4.42	0.69
PFDA	-1.07	-6.58, 4.75	-2.26	-9.54, 5.6	0.35	-7.75, 9.16	0.65
Adiponectin							
PFOS	3.14	-0.47, 6.88	4.23	-0.79, 9.5	2.06	-2.89, 7.25	0.55
PFOA	1.87	-1.65, 5.52	0.70	-3.94, 5.58	3.06	-1.71, 8.06	0.47
PFHxS	2.85	-0.79, 6.61	4.84	-0.53, 10.49	1.25	-3.46, 6.19	0.33
PFNA	1.39	-2.71, 5.66	0.99	-4.82, 7.16	1.74	-3.83, 7.64	0.86
PFDA	1.70	-2.68, 6.28	1.01	-4.82, 7.21	2.52	-3.9, 9.38	0.74
Leptin							
PFOS	6.43	-0.58, 13.95	**10.94**	0.95, 21.92	2.05	-7.2, 12.22	0.21
PFOA	-0.28	-6.79, 6.69	-3.09	-11.49, 6.09	2.64	-6.27, 12.4	0.35
PFHxS	4.64	-2.33, 12.11	8.04	-2.31, 19.49	1.94	-6.96, 11.69	0.39
PFNA	5.01	-2.97, 13.64	9.19	-2.51, 22.29	1.37	-8.98, 12.9	0.35
PFDA	4.84	-3.63, 14.05	5.41	-5.94, 18.13	4.17	-7.97, 17.91	0.89
Leptin receptor							
PFOS	-0.08	-3.43, 3.38	-1.12	-5.68, 3.66	0.98	-3.71, 5.89	0.53
PFOA	2.51	-0.87, 6.01	1.49	-2.98, 6.16	3.55	-1.02, 8.34	0.51
PFHxS	**6.65**	3.10, 10.32	4.09	-0.94, 9.36	**8.79**	4.02, 13.78	0.19
PFNA	0.77	-3.12, 4.82	0.13	-5.38, 5.95	1.35	-3.95, 6.96	0.76
PFDA	1.14	-3.02, 5.48	-0.27	-5.77, 5.56	2.83	-3.32, 9.38	0.47

[a] Models were adjusted for maternal age (years), maternal pre-pregnancy body mass index (under, normal, over, obese), maternal smoking during pregnancy (no, 1-5 cigarettes per day, >5 cigarettes per day), parity (primiparous, multiparous), maternal education (low, medium, high), and child sex.

[b] CI: confidence interval; PFAS: per- and polyfluoroalkyl substance; PFOS: perfluorooctane sulfonate; PFOA: perflourooctanoate; PFHxS: perfluorohexanesulfonic acid; PFNA: perfluorononanoate; PFDA, perfluorodecanoate.

[c] Bolded if p-value < 0.05.

Table S2. Percent change of the serum resistin concentrations at age 9 years per doubling of the serum-PFAS concentrations at birth, ages 18 months, and 5 and 9 years, overall and by sex.

ng/mL	Total cohort			Male			Female			P for interaction
	n	% change	95% CI	n	% change	95% CI	n	% change	95% CI	
PFAS concentrations at birth[a]										
PFOS	358	0.26	-3.12, 3.75	187	1.56	-3.13, 6.47	171	-1.06	-5.68, 3.79	0.44
PFOA	358	1.27	-2.15, 4.81	187	2.88	-1.68, 7.65	171	-0.38	-4.88, 4.34	0.30
PFHxS	358	-2.30	-5.75, 1.26	187	-0.42	-5.45, 4.89	171	-3.86	-8.35, 0.84	0.32
PFNA	358	-2.01	-5.89, 2.02	187	-0.25	-5.85, 5.68	171	-3.57	-8.70, 1.86	0.40
PFDA	358	-2.30	-6.46, 2.05	187	-2.60	-8.11, 3.24	171	-1.93	-8.02, 4.56	0.88
PFAS concentrations at 18 months[a]										
PFOS	277	-2.13	-5.43, 1.28	142	0.15	-4.32, 4.82	135	-3.95	-8.44, 0.77	0.22
PFOA	277	-2.10	-5.36, 1.27	142	-1.17	-5.55, 3.42	135	-3.25	-7.53, 1.23	0.51
PFHxS	277	**-1.73**	-3.33, -0.12	142	-0.99	-3.15, 1.22	135	-2.13	-4.24, 0.04	0.47
PFNA	277	**-4.83**	-7.96, -1.60	142	-3.31	-7.85, 1.45	135	**-5.44**	-9.42, -1.28	0.50
PFDA	277	**-5.15**	-8.96, -1.18	142	-2.22	-7.72, 3.60	135	**-7.40**	-12.29, -2.25	0.18
PFAS concentrations at 5 years[a]										
PFOS	294	-1.12	-5.64, 3.62	154	4.08	-2.87, 11.53	140	-5.48	-10.93, 0.29	0.04
PFOA	294	-1.04	-6.17, 4.37	154	1.48	-5.71, 9.22	140	-5.11	-11.67, 1.93	0.20
PFHxS	294	-1.22	-4.96, 2.68	154	2.81	-2.72, 8.65	140	-4.38	-8.94, 0.42	0.06
PFNA	294	-0.13	-3.47, 3.33	154	-0.50	-5.02, 4.25	140	0.03	-4.58, 4.87	0.88
PFDA	294	0.51	-2.60, 3.72	154	0.38	-3.76, 4.69	140	0.23	-4.24, 4.90	0.96
PFAS concentrations at 9 years[b]										
PFOS	370	-2.76	-6.28, 0.90	192	-2.02	-6.87, 3.08	178	-3.57	-8.59, 1.74	0.67
PFOA	370	1.31	-4.56, 7.54	192	0.50	-7.63, 9.34	178	2.12	-6.12, 11.09	0.79
PFHxS	370	-2.78	-6.86, 1.47	192	-1.93	-7.72, 4.22	178	-3.61	-9.24, 2.36	0.69
PFNA	370	-2.32	-6.16, 1.68	192	-3.30	-8.36, 2.05	178	-1.09	-6.86, 5.04	0.58
PFDA	370	-2.10	-5.82, 1.77	192	-3.07	-7.96, 2.08	178	-0.86	-6.46, 5.08	0.57

[a] Models were adjusted for maternal age (years), maternal pre-pregnancy body mass index (under, normal, over, obese), maternal smoking during pregnancy (no, 1-5 cigarettes per day, >5 cigarettes per day), parity (primiparous, multiparous), maternal education (low, medium, high), and child sex.
[b] Models were adjusted for maternal education (low, medium, high) and child sex.
[c] CI: confidence interval; PFAS: per- and polyfluoroalkyl substance; PFOS: perfluorooctane sulfonate; PFOA: perflourooctanoate; PFHxS: perfluorohexanesulfonic acid; PFNA: perfluorononanoate; PFDA, perfluorodecanoate.
[d] Bolded if p-value < 0.05.

Table S3. Percent change of the serum adiponectin concentrations at age 9 years per doubling of the serum-PFAS concentrations at birth, ages 18 months, and 5 and 9 years, overall and by sex.

ng/mL	Total cohort			Male			Female			P for interaction
	n	% change	95% CI	n	% change	95% CI	n	% change	95% CI	
PFAS concentrations at birth[a]										
PFOS	358	1.18	-2.17, 4.64	187	1.60	-2.02, 5.35	171	1.05	-3.58, 5.91	0.94
PFOA	358	1.34	-2.01, 4.82	187	0.49	-5.23, 6.56	171	0.60	-3.87, 5.28	0.64
PFHxS	358	-2.21	-5.60, 1.29	187	2.16	-2.05, 6.55	171	-2.51	-6.98, 2.18	0.85
PFNA	358	1.02	-2.91, 5.11	187	0.34	-3.54, 4.37	171	-0.80	-5.99, 4.68	0.33
PFDA	358	-0.72	-4.88, 3.62	187	2.07	-1.74, 6.04	171	-3.08	-8.98, 3.21	0.31
PFAS concentrations at 18 months[a]										
PFOS	277	0.43	-2.98, 3.95	142	-2.29	-6.65, 2.28	135	3.74	-1.11, 8.84	0.08
PFOA	277	1.54	-1.85, 5.04	142	-0.44	-4.87, 4.19	135	3.03	-1.54, 7.82	0.30
PFHxS	277	0.24	-1.40, 1.91	142	-0.97	-3.15, 1.26	135	1.22	-0.98, 3.48	0.17
PFNA	277	-0.89	-4.2, 2.54	142	-2.78	-7.40, 2.09	135	0.84	-3.47, 5.34	0.27
PFDA	277	-1.54	-5.55, 2.64	142	-1.94	-7.54, 4.00	135	-0.72	-6.04, 4.90	0.76
PFAS concentrations at 5 years[a]										
PFOS	294	0.67	-3.51, 5.04	154	0.34	-5.84, 6.91	140	0.53	-4.80, 6.16	0.96
PFOA	294	-1.20	-5.85, 3.69	154	-1.97	-8.34, 4.84	140	-0.38	-6.69, 6.36	0.74
PFHxS	294	2.13	-1.38, 5.76	154	0.53	-4.43, 5.76	140	2.28	-2.20, 6.97	0.62
PFNA	294	1.02	-2.05, 4.18	154	1.02	-3.18, 5.39	140	0.46	-3.77, 4.87	0.86
PFDA	294	0.52	-2.31, 3.43	154	1.04	-2.77, 4.98	140	-0.55	-4.60, 3.66	0.59
PFAS concentrations at 9 years[b]										
PFOS	370	1.60	-2.02, 5.35	192	3.80	-1.24, 9.11	178	-0.80	-5.88, 4.56	0.22
PFOA	370	0.49	-5.23, 6.56	192	1.30	-6.75, 10.05	178	-0.31	-8.22, 8.28	0.79
PFHxS	370	2.16	-2.05, 6.55	192	4.86	-1.22, 11.31	178	-0.41	-6.12, 5.64	0.23
PFNA	370	0.34	-3.54, 4.37	192	1.27	-3.95, 6.78	178	-0.81	-6.50, 5.24	0.61
PFDA	370	2.07	-1.74, 6.04	192	4.00	-1.15, 9.42	178	-0.31	-5.84, 5.55	0.28

[a] Models were adjusted for maternal age (years), maternal pre-pregnancy body mass index (under, normal, over, obese), maternal smoking during pregnancy (no, 1-5 cigarettes per day, >5 cigarettes per day), parity (primiparous, multiparous), maternal education (low, medium, high), and child sex.

[b] Models were adjusted for maternal education (low, medium, high) and child sex.

[c] CI: confidence interval; PFAS: per- and polyfluoroalkyl substance; PFOS: perfluorooctane sulfonate; PFOA: perflourooctanoate; PFHxS: perfluorohexanesulfonic acid; PFNA: perfluorononanoate; PFDA, perfluorodecanoate.

[d] Bolded if p-value < 0.05.

Table S4. Percent change of the serum leptin concentrations at age 9 years per doubling of the serum-PFAS concentrations at birth, ages 18 months, and 5 and 9 years, overall and by sex.

ng/mL	Total cohort			Male			Female			P for interaction
	n	% change	95% CI	n	% change	95% CI	n	% change	95% CI	
PFAS concentrations at birth[a]										
PFOS	358	4.03	-5.46, 14.48	187	7.28	-5.98, 22.42	171	0.81	-11.79, 15.21	0.51
PFOA	358	6.77	-2.98, 17.51	187	6.59	-6.09, 20.98	171	6.96	-6.00, 21.71	0.97
PFHxS	358	5.55	-4.52, 16.69	187	-2.87	-15.97, 12.27	171	13.21	-0.92, 29.35	0.12
PFNA	358	4.19	-6.94, 16.65	187	10.21	-6.22, 29.52	171	-0.92	-14.97, 15.45	0.34
PFDA	358	-0.84	-12.21, 12.00	187	2.85	-12.61, 21.05	171	-5.15	-20.71, 13.47	0.51
PFAS concentrations at 18 months[a]										
PFOS	277	**-9.91**	-18.00, -1.02	142	-7.94	-18.98, 4.61	135	**-13.33**	-24.21, -0.88	0.52
PFOA	277	-8.13	-16.30, 0.84	142	-6.17	-17.37, 6.54	135	**-12.55**	-22.97, -0.72	0.44
PFHxS	277	-3.90	-8.15, 0.55	142	-4.41	-10.18, 1.73	135	-4.66	-10.36, 1.40	0.95
PFNA	277	**-12.03**	-19.79, -3.51	142	**-13.72**	-24.63, -1.24	135	**-13.01**	-22.92, -1.82	0.93
PFDA	277	**-11.74**	-21.23, -1.11	142	-9.98	-23.55, 6.00	135	-16.44	-28.30, -2.62	0.51
PFAS concentrations at 5 years[a]										
PFOS	294	-8.22	-19.29, 4.37	154	-5.36	-22.03, 14.86	140	-9.62	-23.46, 6.73	0.72
PFOA	294	**-21.81**	-32.28, -9.73	154	**-20.98**	-35.43, -3.31	140	**-21.74**	-35.71, -4.73	0.95
PFHxS	294	-2.71	-12.53, 8.20	154	-8.64	-21.75, 6.67	140	-0.02	-12.83, 14.67	0.39
PFNA	294	-5.37	-13.81, 3.90	154	-7.63	-18.84, 5.13	140	-2.32	-14.34, 11.39	0.55
PFDA	294	-3.95	-11.89, 4.72	154	-4.84	-15.37, 6.99	140	-3.01	-14.57, 10.13	0.83
PFAS concentrations at 9 years[b]										
PFOS	370	-7.61	-16.87, 2.67	192	-9.21	-21.49, 4.99	178	-5.80	-19.2, 9.81	0.73
PFOA	370	**-26.22**	-37.64, -12.70	192	-20.43	-37.23, 0.87	178	**-31.57**	-46.00, -13.28	0.38
PFHxS	370	-9.85	-20.24, 1.90	192	-13.01	-26.90, 3.51	178	-6.65	-21.39, 10.85	0.57
PFNA	370	**-12.17**	-21.65, -1.54	192	-11.70	-24.26, 2.95	178	-12.76	-26.50, 3.54	0.92
PFDA	370	-7.89	-17.56, 2.91	192	-8.66	-21.24, 5.92	178	-6.92	-21.19, 9.95	0.87

[a] Models were adjusted for maternal age (years), maternal pre-pregnancy body mass index (under, normal, over, obese), maternal smoking during pregnancy (no, 1-5 cigarettes per day, >5 cigarettes per day), parity (primiparous, multiparous), maternal education (low, medium, high), and child sex.
[b] Models were adjusted for maternal education (low, medium, high) and child sex.
[c] CI: confidence interval; PFAS: per- and polyfluoroalkyl substance; PFOS: perfluorooctane sulfonate; PFOA: perflourooctanoate; PFHxS: perfluorohexanesulfonic acid; PFNA: perfluorononanoate; PFDA, perfluorodecanoate.
[d] Bolded if p-value < 0.05.

Table S5. Percent change of the serum leptin receptor concentrations at age 9 years per doubling of the serum-PFAS concentrations at birth, ages 18 months, and 5 and 9 years, overall and by sex.

ng/mL	Total cohort			Male			Female			P for interaction
	n	% change	95% CI	n	% change	95% CI	n	% change	95% CI	
PFAS concentrations at birth[a]										
PFOS	358	**-3.89**	-6.86, -0.83	187	**-6.09**	-10.06, -1.95	171	-1.58	-5.78, 2.81	0.13
PFOA	358	**-3.30**	-6.30, -0.20	187	-2.68	-6.66, 1.46	171	-3.93	-7.93, 0.24	0.65
PFHxS	358	-1.75	-4.95, 1.56	187	-1.67	-6.28, 3.17	171	-1.82	-6.06, 2.62	0.96
PFNA	358	-2.61	-6.18, 1.08	187	-4.37	-9.32, 0.86	171	-1.02	-5.89, 4.10	0.35
PFDA	358	-1.83	-5.70, 2.20	187	-3.15	-8.22, 2.20	178	-0.21	-5.94, 5.87	0.46
PFAS concentrations at 18 months[a]										
PFOS	277	-0.05	-3.15, 3.15	142	0.76	-3.49, 5.19	135	-0.41	-4.81, 4.20	0.72
PFOA	277	-0.40	-3.46, 2.75	142	1.38	-2.86, 5.80	135	-1.23	-5.35, 3.08	0.40
PFHxS	277	0.22	-1.29, 1.74	142	0.95	-1.14, 3.08	135	-0.28	-2.32, 1.80	0.41
PFNA	277	3.02	-0.12, 6.26	142	**5.43**	0.75, 10.32	135	1.62	-2.42, 5.84	0.24
PFDA	277	**4.95**	1.07, 8.98	142	**6.96**	1.30, 12.94	135	3.84	-1.32, 9.27	0.44
PFAS concentrations at 5 years[a]										
PFOS	294	2.34	-1.92, 6.79	154	2.49	-3.93, 9.35	140	0.58	-4.86, 6.32	0.66
PFOA	294	**9.57**	4.50, 14.88	154	**12.29**	5.02, 20.07	140	6.31	-0.4, 13.47	0.25
PFHxS	294	1.24	-2.26, 4.86	154	3.74	-1.48, 9.24	140	-1.28	-5.69, 3.33	0.16
PFNA	294	0.25	-2.81, 3.41	154	-1.26	-5.44, 3.10	140	0.81	-3.52, 5.33	0.51
PFDA	294	1.44	-1.42, 4.38	154	0.45	-3.40, 4.46	140	1.86	-2.36, 6.27	0.64
PFAS concentrations at 9 years[b]										
PFOS	370	2.60	-0.91, 6.24	192	2.49	-2.31, 7.53	178	2.73	-2.34, 8.06	0.95
PFOA	370	4.29	-1.42, 10.32	192	2.27	-5.54, 10.72	178	6.34	-1.77, 15.11	0.50
PFHxS	370	4.10	-0.02, 8.38	192	**6.96**	1.02, 13.26	178	1.37	-4.20, 7.26	0.19
PFNA	370	2.90	-0.92, 6.87	192	1.54	-3.48, 6.83	178	4.63	-1.14, 10.73	0.44
PFDA	370	3.50	-0.21, 7.35	192	3.55	-1.38, 8.72	178	3.44	-2.08, 9.27	0.98

[a] Models were adjusted for maternal age (years), maternal pre-pregnancy body mass index (under, normal, over, obese), maternal smoking during pregnancy (no, 1-5 cigarettes per day, >5 cigarettes per day), parity (primiparous, multiparous), maternal education (low, medium, high), and child sex.
[b] Models were adjusted for maternal education (low, medium, high) and child sex.
[c] CI: confidence interval; PFAS: per- and polyfluoroalkyl substance; PFOS: perfluorooctane sulfonate; PFOA: perflourooctanoate; PFHxS: perfluorohexanesulfonic acid; PFNA: perfluorononanoate; PFDA, perfluorodecanoate.
[d] Bolded if p-value < 0.05.

Table S6. Posterior inclusion probabilities (PIPs) of five PFASs measured at birth in relation to the serum-adipokine concentrations at birth in the overall population.

PFAS	Resistin	Adiponectin	Leptin	Leptin receptor
PFOS	0.049	0.180	0.221	0.083
PFOA	0.014	0.007	0.032	0.019
PFHxS	0.009	0.011	0.121	0.821
PFNA	0.061	0.010	0.069	0.006
PFDA	0.031	0.003	0.035	0.010

Table S7. Posterior inclusion probabilities (PIPs) of five PFASs measured at birth, 18 months, and 5 and 9 years in relation to the serum-adipokine concentrations at 9 years in the overall population.

PFAS	Resistin	Adiponectin	Leptin	Leptin receptor
PFAS concentrations at birth				
PFOS	0.023	0.074	0.068	0.459
PFOA	0.042	0.009	0.651	0.036
PFHxS	0.058	0.014	0.088	0.004
PFNA	0.105	0.022	0.166	0.009
PFDA	0.299	0.012	0.171	0.013
PFAS concentrations at 18 months				
PFOS	0.171	0.012	0.173	0.116
PFOA	0.174	0.012	0.182	0.041
PFHxS	0.138	0.009	0.299	0.028
PFNA	0.573	0.011	0.641	0.078
PFDA	0.248	0.013	0.327	0.198
PFAS concentrations at 5 years				
PFOS	0.022	0.006	0.068	0.007
PFOA	0.025	0.012	0.904	0.880
PFHxS	0.025	0.008	0.044	0.009
PFNA	0.066	0.005	0.031	0.009
PFDA	0.110	0.005	0.039	0.014
PFAS concentrations at 9 years				
PFOS	0.084	0.074	0.123	0.010
PFOA	0.022	0.007	0.925	0.031
PFHxS	0.037	0.007	0.047	0.017
PFNA	0.050	0.031	0.140	0.009
PFDA	0.039	0.147	0.103	0.030
